# Protective Effects of Lentinan Against Lipopolysaccharide-Induced Mastitis in Mice

**DOI:** 10.3389/fphar.2021.755768

**Published:** 2021-09-24

**Authors:** Huifang Yin, Guanhong Xue, Ailing Dai, Haichong Wu

**Affiliations:** ^1^ College of Life Sciences of Longyan University, Longyan, China; ^2^ Fujian Provincial Key Laboratory for the Prevention and Control of Animal Infectious Diseases and Biotechnology, Longyan, China; ^3^ Key Laboratory of Preventive Veterinary Medicine and Biotechnology, Longyan University, Longyan, China; ^4^ Department of Veterinary Medicine, College of Animal Sciences, Zhejiang University, Hangzhou, China

**Keywords:** lentinan, mastitis, LPS, inflammation, Wnt/β-catenin pathway

## Abstract

Mastitis is a worldwide production disease in dairy cows, which mainly affects milk yield, causing huge economic losses to dairy farmers. Lentinan is a kind of polysaccharide extracted from *Lentinus edodes*, which has no toxicity and possesses various pharmacological activities including antibacterial and immunomodulatory effects. Therefore, the anti-inflammatory function of lentinan on LPS-stimulated mastitis was carried out, and the mechanism involved was explored. *In vivo*, lentinan greatly reduced LPS-stimulated pathological injury, myeloperoxidase (MPO) activity, and the proinflammatory factor production (TNF-α and IL-1β) in mice. Further study was performed to determine the activation of the Wnt/β-catenin pathway during LPS stimulation. These results suggested that LPS-induced activation of the Wnt/β-catenin pathway was suppressed by lentinan administration. *In vitro*, we observed that the mouse mammary epithelial cell (mMEC) viability was not affected by lentinan treatment. As expected, LPS increased the TNF-α and IL-1β protein secretion and the activation of the Wnt/β-catenin pathway that was inhibited by lentinan administration in a dose-dependent manner in mMECs. Conclusively, lentinan exerts the anti-inflammatory function in LPS-stimulated mastitis *via* inhibiting the activation of the Wnt/β-catenin pathway. Thus, the results of our study also gave an insight that lentinan may serve as a potential treatment for mastitis.

## Introduction

Mastitis, one of the most prevalent diseases in dairy cows, is mainly characterized by the inflammation of the mammary gland with major economic, hygienic, and welfare implications (Wu et al., 2016; Dai et al., 2019; Puggioni et al., 2019). There are abundant pathogenic microorganisms that can cause mastitis, such as Gram-negative bacteria *Escherichia coli* (Zadoks et al., 2011; Jiang et al., 2018). *Escherichia coli* inflicts widespread infection in humans and is one of the most common causative pathogens in bovine mastitis (Filioussis et al., 2020).

Lipopolysaccharide (LPS, which is also called the endotoxin), a main constituent of the Gram-negative bacterial cell wall, has been often used to mimic *E. coli*-infected mastitis *in vivo* as well as in cultured mammary epithelial cells (Chen et al., 2018; Kusebauch et al., 2018). When the components of pathogens (for example LPS) or their pathogen-associated molecular patterns are recognized by the innate immune system, multiple signaling pathways will be initiated to eradicate infection and protect the host against pathogens (Stokes et al., 2015; Iida et al., 2018; Kumar, 2019). Increasing evidence has revealed that the Wnt/β-catenin signaling pathway is involved in several inflammatory diseases (Mu et al., 2020; Quandt et al., 2021; Zhou et al., 2021). Therefore, pharmacological inhibition or interference of the Wnt/β-catenin pathway may be an effective strategy for treatment of several inflammatory diseases.

At present, antibiotics are the major drugs for the treatment of mastitis, but the emergence of antibiotic resistance has brought great trouble, threatened the health of humans and animals, and even caused the possibility of zoonotic bacteria entering the food chain (Doehring and Sundrum, 2019; Meade et al., 2019). Lentinan, a plant polysaccharide extracted from the mushroom, has harmless and few side effects on the human body (Wang X. et al., 2019; Zhang and Zhao, 2019). Although lentinan has been reported to possess various pharmacological activities such as anticancer, antibacterial, antiviral, and antioxidant effects (Nishitani et al., 2013; Wang X. et al., 2019; Hou et al., 2020), the potential protective mechanisms of lentinan on LPS-induced mastitis remain to be explored.

We hypothesized that lentinan alleviated LPS-induced mastitis by interfering with the activation of the Wnt/β-catenin pathway, which may also be a potential target for treatment of bovine mastitis and other inflammatory diseases. In the present research, the LPS-induced mouse mastitis was used to evaluate whether lentinan could protect the LPS-stimulated mastitis and explain its therapeutic mechanisms.

## Materials and Methods

### Reagents

Lentinan was obtained from Shanghai Yuanye Biotechnology Co., Ltd., and dissolved with DMSO to prepare a final concentration of 100 mg/ml. When lentinan is used, it is diluted to the experimental concentrations (DMSO<0.1%). LPS was purchased from Sigma Chemical CO (St. Louis, United States). A mouse myeloperoxidase (MPO) ELISA kit was obtained from MultiSciences (Lianke) Biotech Co., Ltd (Zhejiang, China).

### Animal Treatment and Experimental Groups

Mice were purchased from the Laboratory Animal Center of Zhejiang University (Hangzhou, China). Ninety BALB/c female mice (8 week old) were used in this experiment. Food and water were available *ad libitum*. The mice were kept in separate cages for a 12 h dark light cycle under controlled temperature (24°C ± 1°C) and 60% humidity for 1 week before the research. All experimental procedures and protocols were approved by the Institutional Animal Care and Use Committee in Zhejiang University.

The mice were randomly classified into six groups, each comprising fifteen mice: Control group, LPS group, lentinan (5, 10, and 20 mg/kg) + LPS groups, and dexamethasone group (5 mg/kg). The mastitis model was carried out as described previously by us (Xingxing et al., 2018). In brief, 100 μl of LPS (1 mg/ml) was infused into two abdominal mammary glands (R4 and L4) in mice under anesthesia with pentobarbital. Mice received an intraperitoneal injection (ip) of different lentinan concentrations (5, 10, and 20 mg/kg) or dexamethasone after 1 h of LPS or saline ip treatment. After 24 h, the mice were sacrificed by CO_2_ inhalation at the same time. The mouse mammary tissues were collected and stored at −80°C until being analyzed.

### Histopathologic Evaluation of the Mammary Tissues

The mouse mammary gland tissues were excised and fixed in 10% formalin for subsequent histopathological analysis. In brief, tissues were dehydrated with different concentrations of alcohol, paraffin-embedded sections were prepared at a 4-µm thickness, and hematoxylin and eosin (H and E) staining was then performed to observe the morphology changes with an optical microscope (Olympus, Japan).

### Myeloperoxidase (MPO) Analysis

MPO activity in mammary gland tissue was detected in tissue homogenates prepared as described above using the ELISA kit following the instruction book of the producer. In addition, mammary tissues were fixed in 4% paraformaldehyde, embedded in paraffin, sectioned, and then incubated with the MPO antibody (Servicebio, China). Immunopositive cells were counted, and positive cells in mammary gland tissue sections were quantified to the tissue area.

### Cell Culture and Treatment

Epithelial cells from the mammary gland tissue of lactating mice were cultured as described previously (Wu et al., 2017). The mouse mammary epithelial cells (mMECs) were cultured in DMEM containing 10% FBS, 100 U/mL penicillin–streptomycin, and 10 μg/ml insulin in a 5% CO_2_ incubator. The cells were pretreated with different concentrations of lentinan (5, 10, and 20 μg/ml) or dexamethasone for 1 h before LPS challenge.

### Cell Biological Examination and MTT Assay

The cells were fixed with paraformaldehyde at room temperature for 15 min and then washed three times with PBS in a twelve-well plate. Next, the cells were sealed with 10% normal goat serum at room temperature for 1 , followed by incubation with the primary antibody CK-18 at 4°C for 12 h. The cells were then incubated with the fluorescent-labeled secondary antibody (Bioss, China) for 45 min at room temperature and washed three times in PBS. Finally, DAPI was used to stain the cell nuclei, which were then observed using a laser scanning confocal microscope (Leica, Germany).

The mMEC viability was evaluated by the 3-[4,5-dimethylthiazol-2-yl]-2,5 diphenyl tetrazolium bromide (MTT) experiment. Cells (1 × 10^5^ cell/mL) were cultured in 96-well plates for 6 hours. The cells were stimulated with lentinan (5, 10, and 20 μg/ml) for 24 h. Next, the MTT (5 mg/ml) agent was added in mMECs for 4 h, and 100 μl of DMSO per well was added. The optical density (OD) at 570 nm was read with a microplate reader (Thermo, United States).

### Immunofluorescence Staining

Immunofluorescence staining assay of the mouse mammary tissue and mMECs was carried out. Briefly, the mouse mammary tissues or cells were fixed in 10% formalin, and tissues were embedded in paraffin. The tissue or cell slice was permeated with PBS appending Triton X-100 (0.3%, Sigma, United States) and 10% BSA. The tissue or cell slice was hatched for 12 h at 4°C with a special antibody for Wnt3a and *β*-catenin (Servicebio, China) and a Cy3 secondary antibody. Then, Wnt3a and *β*-catenin proteins were determined and immobilized using mounting media supplemented with DAPI. Finally, all of the slices were observed with fluorescence microscopy.

### Cytokine Assay

The cytokine secretion after LPS challenge was assessed with the ELISA kit in the mouse mammary tissues and mMECs. The tissues were homogenized in ice-cold PBS and then centrifugation at 10,000 rpm, 4°C for 15 min. Harvested tissue and cell supernate to detect the production of cytokines (IL-1β and TNF-α) using the ELISA kit following the instruction book of the producer. Finally, the optical density (OD) at 450 nm is read with a microplate reader.

### Western Blot Assay

The total protein of mMECs was obtained by the lysis solution containing the phosphatase repressor. The BCA kit was used to determine the protein concentration. Then, samples with the same amount of protein were applied to 10% SDS-PAGE gel electrophoresis and then transferred to the PVDF membrane. After being placed in the 5% skim milk, the membrane was washed three times and incubated with the corresponding primary antibody at 4°C for 12 h. Next, the membrane was incubated with the secondary antibody at room temperature for 1 hour. The protein band density was detected using a chemiluminescence system.

### Statistical Analysis

SPSS software was used for data analysis. Statistical data were expressed as the mean ± S.E.M. of three individual experiments. Data were analyzed by Student’s *t*-test or one-way analysis of variance (ANOVA). *p* ≤ 0.05 was deemed a statistically significant difference.

## Results

### Effects of Lentinan on LPS-Induced Histopathological Changes

**FIGURE 1:**
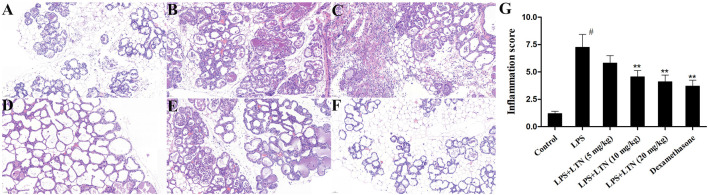
Effects of lentinan on LPS-stimulated histopathological changes. Histopathological changes in mammary gland tissues (H and E). **(A)** Control group, **(B)** LPS group, **(C–E)** lentinan (5, 10, and 20 mg/kg) groups, **(F)** dexamethasone group, and **(G)** histopathological grade score. The blue arrow indicates the mammary gland tissue lesion area. All data are represented as the mean ± S.E.M. of three replicates. ^#^ *p* < 0.05 vs the control group. *p < 0.05 vs. the LPS group. **p < 0.01 compared with the LPS group. ***p < 0.01 compared with the LPS group.

The histological analysis was used to evaluate the mouse mammary tissue damage. Histological analysis showed that the mammary tissue of the control group was intact without histopathological changes (Figure 1A). In the LPS group, the injury of mouse mammary tissue was obvious, and inflammatory cell infiltration was extensive and hyperemia (Figure 1B). However, the extensive inflammatory cell infiltration and hyperemia were relieved, and the tissue structure was relatively complete in lentinan or dexamethasone treatment (Figures 1C–F). Moreover, in order to further verify the degree of tissue damage, the histopathological changes of the mouse mammary gland were evaluated according to the number of infiltrated inflammatory cells. As described in the literature, the pathological grade was scored according to 0–5 (Xingxing et al., 2018). As expected, the result was consistent with pathological sections (Figure 1G).

### Effects of Lentinan on Myeloperoxidase Activity

**FIGURE 2:**
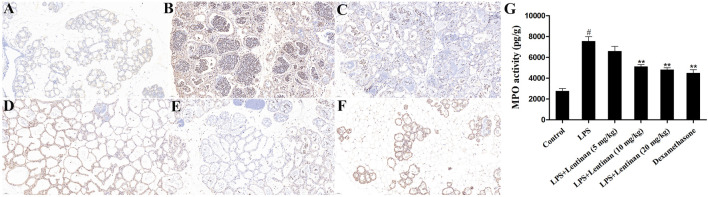
Effects of lentinan on MPO activity. **(A,B)** MPO activity assay in lentinan-treated mMECs. All data are represented as the mean ± S.E.M. of three replicates. *p < 0.05 vs. the LPS group. **p < 0.01 compared with the LPS group. ***p < 0.01 compared with the LPS group.

MPO is an enzyme in neutrophils, and its activity is related to neutrophil infiltration (Lin et al., 2020). As displayed in Figure 2A, compared with the control group, the MPO activity was obviously enhanced in LPS challenge. Lentinan treatment reduced MPO activity in a dose-dependent manner, especially at high concentration. In order to further verify the effect of Lentinan on MPO activity, the immunofluorescence technique was performed. As expected, LPS-enhanced MPO activity was decreased by lentinan or dexamethasone treatment (Figure 2B).

### Lentinan Inhibited the Secretion of Proinflammatory Factors

**FIGURE 3:**
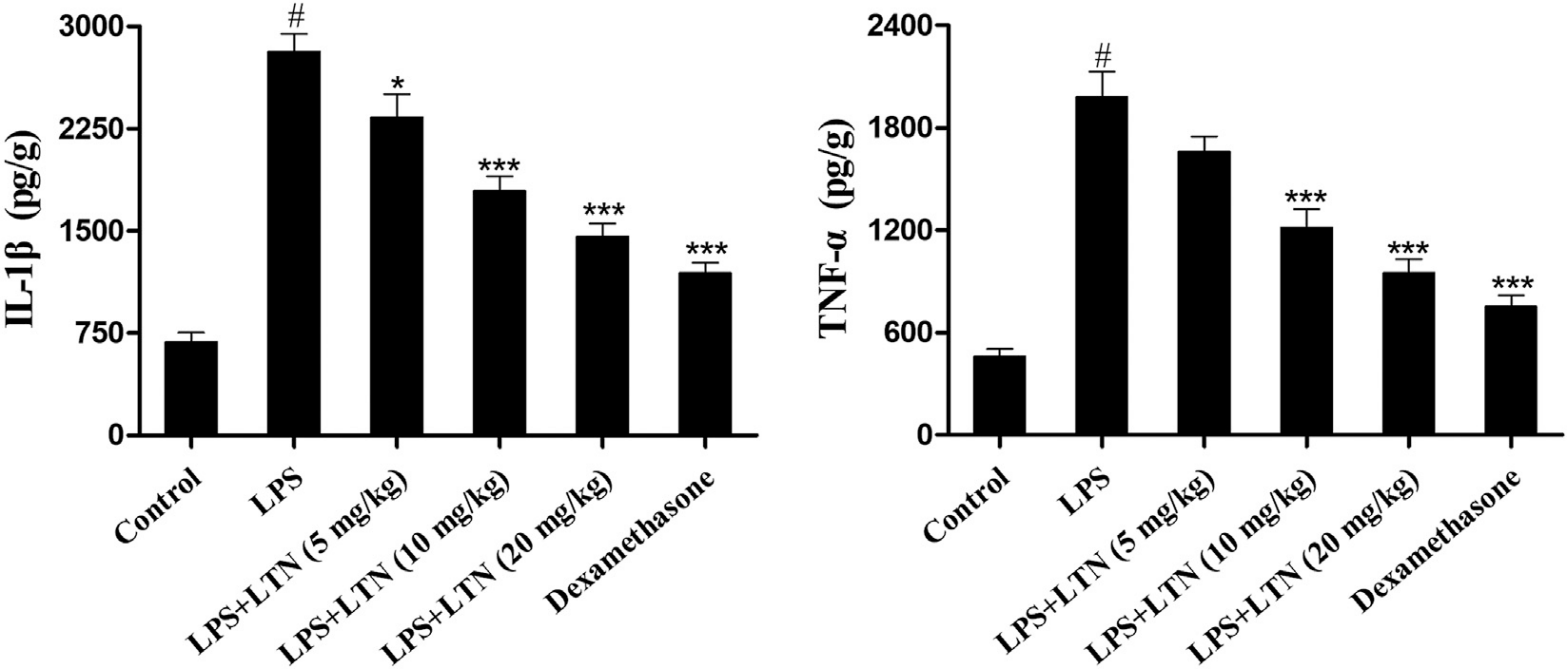
Lentinan inhibited the secretion of proinflammatory factors. The expression of TNF-α and IL-1β in LPS-stimulated mouse mastitis was detected using ELISA kits. All data are represented as the mean ± S.E.M. of three replicates. *p < 0.05 vs the LPS group. **p < 0.01 compared with the LPS group. ***p < 0.01 compared with the LPS group.

The secretion of proinflammatory factors in mouse mammary tissues was detected using ELISA kits. The result of ELISA assay displayed that LPS markedly promoted the production of TNF-α and IL-1β. In contrast, lentinan or dexamethasone treatment substantially decreased the levels of TNF-α and IL-1β (Figure 3). These abovementioned results suggested that lentinan significantly inhibited the secretion of proinflammatory factors in LPS-induced mastitis at a concentration of 20 mg/kg. Thus, this concentration of lentinan was used to study the protective mechanism of LPS-induced mastitis.

### Effects of Lentinan on the Activation of the Wnt/β-Catenin Pathway

**FIGURE 4:**
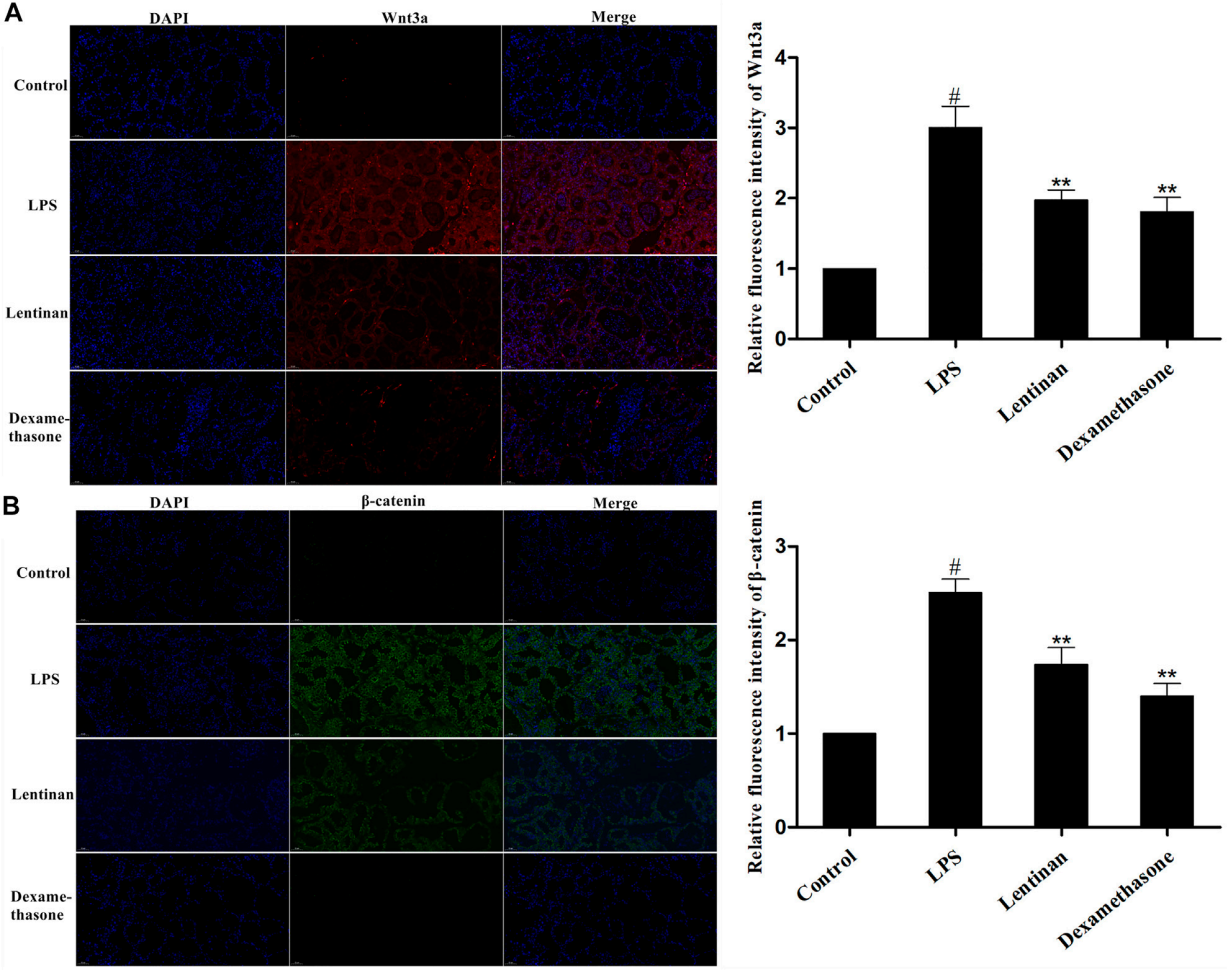
Effects of lentinan on the activation of the Wnt/β-catenin pathway. The activation of the Wnt/β-catenin pathway in LPS-stimulated mouse mastitis was determined by immunofluorescence assay. All data are represented as the mean ± S.E.M. of three replicates.

The Wnt/β-catenin signaling pathway is an evolutionarily conserved mechanism that is fundamentally vital for inflammation-related diseases (Guan et al., 2021). We evaluated whether lentinan alleviated the LPS-induced inflammatory response by suppressing the Wnt/β-catenin pathway. The result of immunofluorescence assay displayed that LPS treatment significantly enhanced the activation of the Wnt/β-catenin signaling pathway that was reduced by lentinan treatment (Figure 4).

### Cell Biological Detection

**FIGURE 5:**
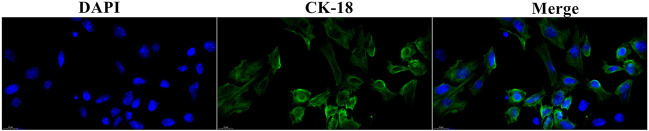
Cell biological detection. The nucleus was dyed blue. The cytoplasm was dyed green by CK-18.

CK-18 is commonly used to identify the integrity of epithelial cells. Thus, mMECs were pretreated with the blue fluorescent pigment to identify the cell nucleus and CK-18 labeled with the green fluorescent pigment to show the cell integrity. The result is displayed in Figure 5.

### Effects of Lentinan on Inflammatory Response of Mouse Mammary Epithelial Cells

**FIGURE 6:**
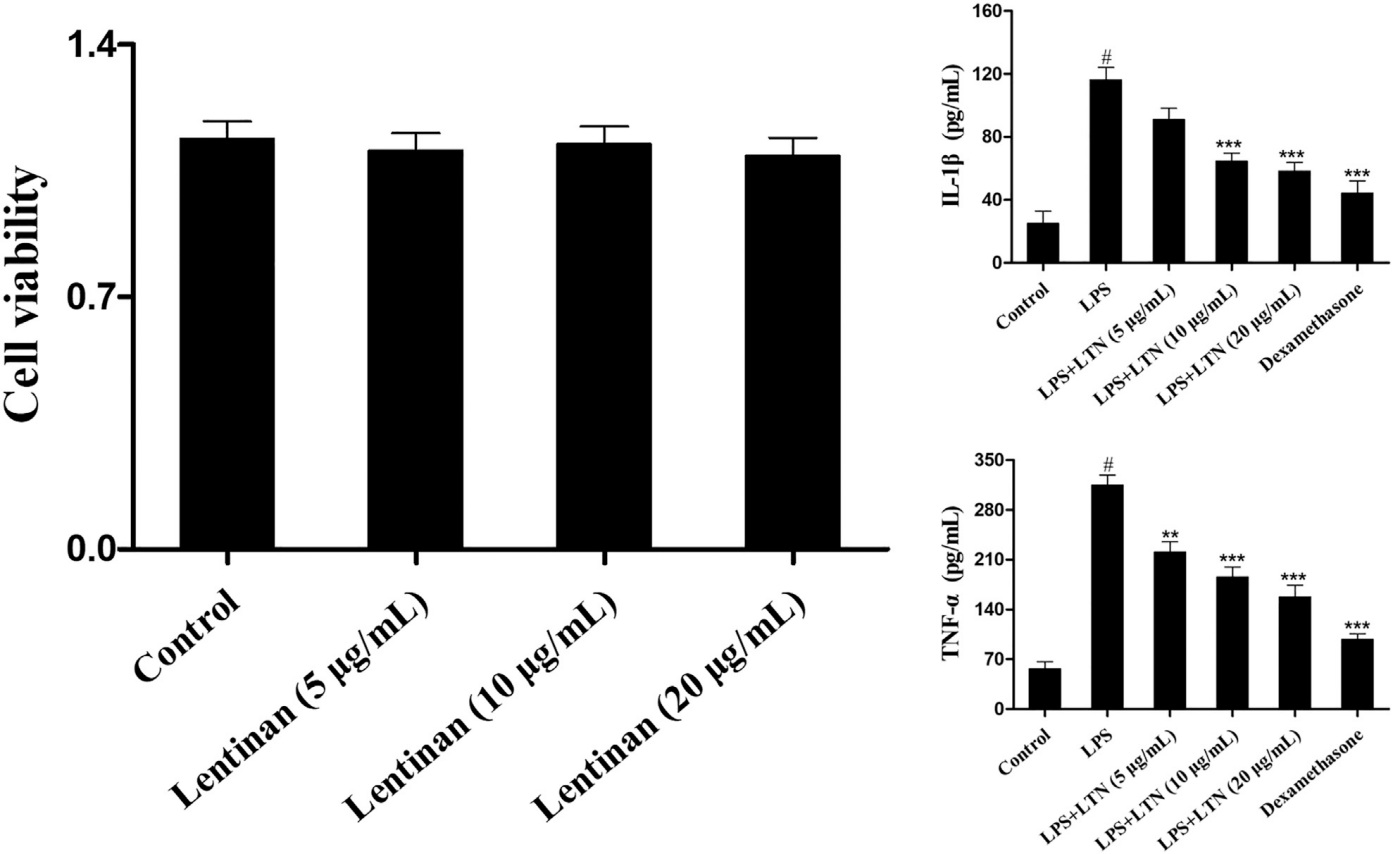
Effects of lentinan on inflammatory response of LPS-induced mouse mammary epithelial cells. **(A)** The potential cytotoxicity of lentinan (5, 10, and 20 μg/ml) on mMECs was detected by MTT experiment. **(B)** The TNF-α and IL-1β protein levels were detected using the ELISA kit in LPS-stimulated mMECs. All data are represented as the mean ± S.E.M. of three replicates. *p < 0.05 vs. the LPS group. **p < 0.01 compared with the LPS group. ***p < 0.01 compared with the LPS group.

First, the potential cytotoxicity of lentinan on mMECs was detected by MTT experiment. As shown in Figure 6A, the cell viability was not affected by lentinan treatment. To investigate the effects of lentinan on inflammatory response in LPS-stimulated mMECs, the TNF-α and IL-1β protein levels were detected using the ELISA kit. As displayed in Figure 6B, LPS increased TNF-α and IL-1β secretion that was inhibited by lentinan or dexamethasone administration.

### Effects of Lentinan on the Activation of the Wnt/β-Catenin Pathway in mMECs

**FIGURE 7:**
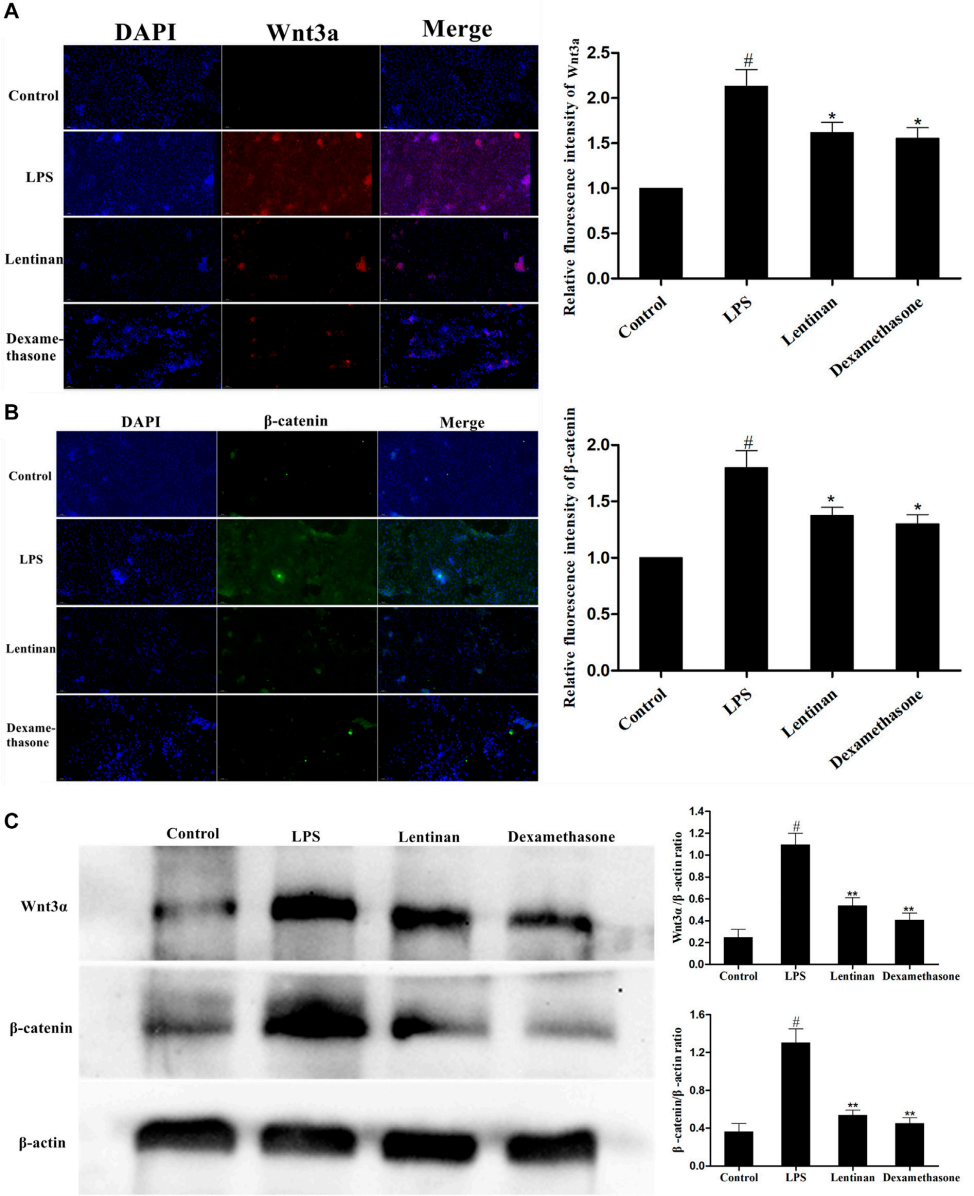
Effects of lentinan on the activation of the Wnt/β-catenin pathway in mMECs. **(A,B)** The activation of the Wnt/β-catenin signaling pathway in LPS-stimulated mMECs was also determined by the immunofluorescence technique. **(C)** The expression of Wnt/β-catenin proteins was detected by western blot. *β*-actin served as an internal control. All data are represented as the mean ± S.E.M. of three replicates.

The activation of the Wnt/β-catenin signaling pathway in LPS-stimulated mMECs was also determined by immunofluorescence assay. The result showed that LPS challenge greatly enhanced the activation of the Wnt/β-catenin pathway, but that was reduced by lentinan treatment or dexamethasone administration (Figures 7A,B). In order to confirm the effect of lentinan on the Wnt/β-catenin pathway activation, western blot was performed in mMECs. Consistent with the results in Figure 7C, the downstream factors in the Wnt/β-catenin pathway, Wnt3α, and *β*-catenin were activated upon LPS challenge, while lentinan or dexamethasone administration downregulated the Wnt3α and *β*-catenin protein expression.

## Discussion

Mastitis is a worldwide production disease of dairy cattle, which mainly affects milk yield, causing huge economic losses to dairy farmers (Krishnamoorthy et al., 2021). It is well known that inflammation is harmful to the breast, but the effect of mastitis on parts other than the breast is not obvious until researchers began to pay attention to environmental pathogens (Ruegg, 2017). The main pathogen causing mastitis in dairy cows is *Escherichia coli* (Shao et al., 2015). At present, antibacterial agents are still the main treatment and prevention of mastitis in most dairy farms (Stevens et al., 2016). However, consumers and public health authorities are increasingly concerned about the use of antibiotics to balance animal health and the development of antimicrobial resistance on farms (Ruegg, 2017; Nobrega et al., 2020). Lentinan is a kind of polysaccharide extracted from *Lentinus edodes*, which has no toxicity and possesses various pharmacological activities such as anticancer, antibacterial, and antiviral effects (Antonelli et al., 2020; Lv et al., 2020; Zi et al., 2020). Thus, the anti-inflammatory function of lentinan on LPS-stimulated mastitis was carried out, and the mechanism involved was explored.

In the present research, LPS challenge significantly enhanced the LPS-induced inflammatory injury that was lightened by lentinan treatment. Additionally, as the first line of defense against microorganisms, epithelial cells are cleared by producing a series of immune reactions (Shin et al., 2010). Therefore, the effect of lentinan on LPS-stimulated mouse mammary epithelial cells (mMECs) was also determined. The MTT test showed that the dose of lentinan used in the study had no cytotoxicity, which was consistent with the results of other studies (Guangming et al., 2018; Zhang and Zhao, 2019). Although inflammatory factors can produce adaptive behavioral response and promote energy conservation to fight infection or recover from injury, excessive proinflammatory factors (such as TNF-α and IL-1β) will cause damage to the body and cause inflammation-related diseases (Wang J. et al., 2019). We found that LPS induced the overproduction of proinflammatory factors that were suppressed by lentinan treatment.

There is increasing evidence that in many activated signaling pathways, the Wnt/β-catenin pathway plays vital role in the process of bacterial infection (Umar, 2012; Li et al., 2021). The proinflammatory stimulation of bacterial infection is a necessary condition for activating the Wnt/β-catenin pathway (Silva-García et al., 2014). For instance, the proinflammatory functions were recorded in Wnt3a-stimulated several cells. Moreover, it has been found that mutations in genes encoding *β*-catenin or other Wnt pathway molecules have been verified in several inflammatory diseases, cancers (Castellone et al., 2009). To further explore the anti-inflammatory mechanism of lentinan, we then investigated the activation of the Wnt/β-catenin pathway in LPS-stimulated mMECs. The result showed that lentinan suppressed the activation of the Wnt/β-catenin pathway in LPS-stimulated mMECs. As we had expected, consistent results were obtained in the tissue immunofluorescence test.

In conclusion, the present results suggested that lentinan had a good anti-inflammatory function in LPS-stimulated mastitis through inhibiting the Wnt/β-catenin signaling pathway. Therefore, the results of our study also gave an insight that lentinan may serve as a potential treatment for mastitis.

## Data Availability

The original contributions presented in the study are included in the article/Supplementary Material, and further inquiries can be directed to the corresponding author.

## References

[B1] Antonelli M. Donelli D. Firenzuoli F. (2020). “Lentinan for Integrative Cancer Treatment: an Umbrella Review,” in 1st International Electronic Conference on Biomolecules: Natural and Bio-Inspired Therapeutics for Human Diseases. 10.3390/iecbm2020-08733

[B2] Castellone M. D. De Falco V. Rao D. M. Bellelli R. Muthu M. Basolo F. (2009). The Beta-Catenin Axis Integrates Multiple Signals Downstream from RET/papillary Thyroid Carcinoma Leading to Cell Proliferation. Cancer Res. 69, 1867–1876. 10.1158/0008-5472.CAN-08-1982 19223551 PMC2746012

[B3] Chen X. Zheng X. Zhang M. Yin H. Jiang K. Dai H. A. (2018). Nuciferine Alleviates LPS-Induced Mastitis in Mice via Suppressing the TLR4-NF-Κb Signaling Pathway. Inflamm. Res. 67, 903–911. 10.1007/s00011-018-1183-2 30145653

[B4] Chen X. Zheng X. Zhang M. Yin H. Jiang K. Wu H. (2018). Nuciferine Alleviates LPS-Induced Mastitis in Mice via Suppressing the TLR4-NF-Κb Signaling Pathway. Inflamm. Res. 67, 903–911. 10.1007/s00011-018-1183-2 30145653

[B5] Dai H. Coleman D. N. Hu L. Martinez-Cortés I. Wang M. Parys C. (2019). Methionine and Arginine Supplementation Alter Inflammatory and Oxidative Stress Responses during Lipopolysaccharide challenge in Bovine Mammary Epithelial Cells *In Vitro* . J. Dairy Sci. 103, 676–689. 10.3168/jds.2019-16631 31733877

[B6] Doehring C. Sundrum A. (2019). The Informative Value of an Overview on Antibiotic Consumption, Treatment Efficacy and Cost of Clinical Mastitis at Farm Level. Prev. Vet. Med. 165, 63–70. 10.1016/j.prevetmed.2019.02.004 30851929

[B7] Filioussis G. Kachrimanidou M. Christodoulopoulos G. Kyritsi M. Hadjichristodoulou C. Adamopoulou M. (2020). Short Communication: Bovine Mastitis Caused by a Multidrug-Resistant, Mcr-1-Positive (Colistin-resistant), Extended-Spectrum β-Lactamase-Producing *Escherichia coli* Clone on a Greek Dairy Farm. J. Dairy Sci. 103 (1), 852–857. 10.3168/jds.2019-17320 31733863

[B8] Guan X. He Y. Wei Z. Shi C. Li Y. Zhao R. (2021). Crosstalk between Wnt/β-Catenin Signaling and NF-Κb Signaling Contributes to Apical Periodontitis. Int. Immunopharmacol 98, 107843. 10.1016/j.intimp.2021.107843 34153668

[B9] Hou C. Chen L. Yang L. Ji X. (2020). An Insight into Anti-Inflammatory Effects of Natural Polysaccharides. Int. J. Biol. Macromol 153, 248–255. 10.1016/j.ijbiomac.2020.02.315 32114173

[B10] Iida T. Yokoyama Y. Wagatsuma K. Hirayama D. Nakase H. (2018). Impact of Autophagy of Innate Immune Cells on Inflammatory Bowel Disease. Cells 8, 1–13. 10.3390/cells8010007 PMC6356773 30583538

[B11] Jiang K. Ma X. Guo S. Zhang T. Zhao G. Wu H. (2018). Anti-Inflammatory Effects of Rosmarinic Acid in Lipopolysaccharide-Induced Mastitis in Mice. Inflammation 41, 437–448. 10.1007/s10753-017-0700-8 29204872

[B12] Krishnamoorthy P. Goudar A. L. Suresh K. P. Roy P. (2021). Global and Countrywide Prevalence of Subclinical and Clinical Mastitis in Dairy Cattle and Buffaloes by Systematic Review and Meta-Analysis. Res. Vet. Sci. 136, 561–586. 10.1016/j.rvsc.2021.04.021 33892366

[B13] Kumar V. (2019). The Complement System, Toll-Like Receptors and Inflammasomes in Host Defense: Three Musketeers' One Target. Int. Rev. Immunol. 38, 131–156. 10.1080/08830185.2019.1609962 31066339

[B14] Kusebauch U. Hernández-Castellano L. E. Bislev S. L. Moritz R. L. Røntved C. M. Bendixen E. (2018). Selected Reaction Monitoring Mass Spectrometry of Mastitis Milk Reveals Pathogen-Specific Regulation of Bovine Host Response Proteins. J. Dairy Sci. 101, 6532–6541. 10.3168/jds.2017-14312 29655560 PMC6502260

[B15] Li S. Zhou C. Zhu Y. Chao Z. Sheng Z. Zhang Y. (2021). Ferrostatin-1 Alleviates Angiotensin II (Ang II)- Induced Inflammation and Ferroptosis in Astrocytes. Int. Immunopharmacol 90, 107179. 10.1016/j.intimp.2020.107179 33278745

[B16] Lin W. Jia D. Fu C. Zheng Y. Lin Z. (2020). Electro-Acupuncture on ST36 and SP6 Acupoints Ameliorates Lung Injury via Sciatic Nerve in a Rat Model of Limb Ischemia-Reperfusion. J. Inflamm. Res. 13, 465–470. 10.2147/JIR.S264093 32904499 PMC7455772

[B17] Lv Q. Gu Y. Qi Y. Liu Z. Ma G. E. Medicine T. (2020). Effects of Lentinan on NF-Κb Activity in the Liver of Burn Rats with Sepsis. Exp. Ther. Med. 20, 2279–2283. 10.3892/etm.2020.8955 32765705 PMC7401944

[B18] Meade E. Savage M. Garvey P. Slattery M. A. Garvey M. (2019). Antibiotic Resistant Zoonotic Pathogens of Bovine Mastitis and Possible Agents of Foodborne Disease. CJMI 5, 1–11. 10.31031/CJMI.2019.02.000550

[B19] Mu Y. R. Zhou M. Y. Cai L. Liu M. M. Li R. (2020). Overexpression of Aquaporin 1 in Synovium Aggravates Rat Collagen-Induced Arthritis Through Regulating β-Catenin Signaling: An *In Vivo* and *In Vitro* Study. J. Inflamm. Res. 13, 701–712. 10.2147/JIR.S271664 33116749 PMC7550268

[B20] Nishitani Y. Zhang L. Yoshida M. Azuma T. Kanazawa K. Hashimoto T. (2013). Intestinal Anti-inflammatory Activity of Lentinan: Influence on IL-8 and TNFR1 Expression in Intestinal Epithelial Cells. PLoS One 8, e62441. 10.1371/journal.pone.0062441 23630633 PMC3632531

[B21] Nobrega D. B. Tang K. L. Caffrey N. P. De Buck J. Cork S. C. Ronksley P. E. (2020). Prevalence of Antimicrobial Resistance Genes and its Association with Restricted Antimicrobial Use in Food-Producing Animals: A Systematic Review and Meta-Analysis. J. Antimicrob. Chemother. 76, 561–575. 10.1093/jac/dkaa443 33146719

[B22] Puggioni G. M. G. Tedde V. Uzzau S. Guccione J. Ciaramella P. Pollera C. (2019). Evaluation of a Bovine Cathelicidin ELISA for Detecting Mastitis in the Dairy Buffalo: Comparison with Milk Somatic Cell Count and Bacteriological Culture. Res. Vet. Sci. 128, 129–134. 10.1016/j.rvsc.2019.11.009 31783263

[B23] Quandt J. Arnovitz S. Haghi L. Woehlk J. Gounari F. Okoreeh M. (2021). Wnt–β-Catenin Activation Epigenetically Reprograms Treg Cells in Inflammatory Bowel Disease and Dysplastic Progression. Nat. Immunol. 22 (4), 471–484. 10.1038/s41590-021-00889-2 33664518 PMC8262575

[B24] Ren G. Xu L. Lu T. Zhang Y. Wang Y. Yin J. (2018). Protective Effects of Lentinan on Lipopolysaccharide Induced Inflammatory Response in Intestine of Juvenile Taimen (Hucho Taimen, Pallas). Int. J. Biol. Macromol 121, 317–325. 10.1016/j.ijbiomac.2018.09.121 30248420

[B25] Ruegg P. L. (2017). A 100-Year Review: Mastitis Detection, Management, and Prevention. J. Dairy Sci. 100, 10381–10397. 10.3168/jds.2017-13023 29153171

[B26] Shao G. Tian Y. Wang H. Liu F. Xie G. (2015). Protective Effects of Melatonin on Lipopolysaccharide-Induced Mastitis in Mice. Int. Immunopharmacol 29, 263–268. 10.1016/j.intimp.2015.11.011 26590117

[B27] Shin H. S. Yoo I. H. Kim Y. J. Lee J. Y. Kim H. B. Jin S. (2010). MKP1 Regulates the Induction of MCP1 by Streptococcus Pneumoniae Pneumolysin in Human Epithelial Cells. Mol. Cell 30, 263–270. 10.1007/s10059-010-0113-0 20803086

[B28] Silva-García O. Valdez-Alarcón J. Izabal-Aguirre B. A. (2014). The Wnt/β-Catenin Signaling Pathway Controls the Inflammatory Response in Infections Caused by Pathogenic Bacteria. Mediators Inflamm. 2014, 310183. 10.1155/2014/310183 25136145 PMC4127235

[B29] Stevens M. Piepers S. Supré K. Dewulf J. De Vliegher S. (2016). Quantification of Antimicrobial Consumption in Adult Cattle on Dairy Herds in Flanders, Belgium, and Associations with Udder Health, Milk Quality, and Production Performance. J. Dairy Sci. 99, 2118–2130. 10.3168/jds.2015-10199 26778315

[B30] Stokes B. A. Yadav S. Shokal U. Smith L. C. Eleftherianos I. (2015). Bacterial and Fungal Pattern Recognition Receptors in Homologous Innate Signaling Pathways of Insects and Mammals. Front. Microbiol. 6, 19. 10.3389/fmicb.2015.00019 25674081 PMC4309185

[B31] Umar S. (2012). Citrobacter Infection and Wnt Signaling. Curr. Colorectal Cancer Rep. 8, 298–306. 10.1007/s11888-012-0143-4 PMC3865928 24358033

[B32] Wang J. Gao Y. Lin F. Han K. Wang X. (2019a). Omentin-1 attenuates lipopolysaccharide (LPS)-induced U937 macrophages activation by inhibiting the TLR4/MyD88/NF-κB signaling. Arch. Biochem. Biophys. 679, 108187. 10.1016/j.abb.2019.108187 31706880

[B33] Wang X. Wang W. Wang L. Yu C. Zhang G. Zhu H. (2019b). Lentinan Modulates Intestinal Microbiota and Enhances Barrier Integrity in a Piglet Model Challenged with Lipopolysaccharide. Food Funct. 10, 479–489. 10.1039/c8fo02438c 30638239

[B34] Wu H. Zhao G. Jiang K. Chen X. Zhu Z. Qiu C. (2016). Puerarin Exerts an Antiinflammatory Effect by Inhibiting NF-kB and MAPK Activation in Staphylococcus Aureus-Induced Mastitis. Phytother Res. 30, 1658–1664. 10.1002/ptr.5666 27335240

[B35] Wu H. Jiang K. Zhang T. Zhao G. Deng G. (2017). Hydroxytyrosol Exerts an Anti-inflammatory Effect by Suppressing Toll-like Receptor 2 and TLR 2 Downstream Pathways in Staphylococcus Aureus-Induced Mastitis in Mice. J. Funct. Foods 35, 595–604. 10.1016/j.jff.2017.06.035

[B36] Zadoks R. N. Middleton J. R. Mcdougall S. Katholm J. Schukken Y. H. (2011). Molecular Epidemiology of Mastitis Pathogens of Dairy Cattle and Comparative Relevance to Humans. J. Mammary Gland Biol. Neoplasia 16, 357–372. 10.1007/s10911-011-9236-y 21968538 PMC3208832

[B37] Zhang S. Zhao Y. (2019). Lentinan Protects Cardiomyocytes against Hypoxia-Induced Injury by Regulation of microRNA-22/Sirt1. Artif. Cell Nanomed Biotechnol 47, 3938–3946. 10.1080/21691401.2019.1666863 31581847

[B38] Zhou M. Y. Cai L. Feng X. W. Mu Y. R. Meng B. Liu F. Y. (2021). Lentivirus-Mediated Overexpression or Silencing of Aquaporin 1 Affects the Proliferation, Migration and Invasion of TNF-α-Stimulated Rheumatoid Arthritis Fibroblast-Like Synoviocytes by Wnt/β-Catenin Signaling Pathway. J. Inflamm. Res. 14, 1945–1957. 10.2147/JIR.S312783 34017191 PMC8131072

[B39] Zi Y. Jiang B. He C. Liu L. (2020). Lentinan Inhibits Oxidative Stress and Inflammatory Cytokine Production Induced by Benzo(a)pyrene in Human Keratinocytes. J. Cosmet. Dermatol. 19, 502–507. 10.1111/jocd.13005 31135098

