# Supplementary figures and images for: Protective Effects of Lentinan Against Lipopolysaccharide-Induced Mastitis in Mice
Source: Front Pharmacol. 2021 Sep 24;12:755768. doi: 10.3389/fphar.2021.755768 (PMC8497700; doi:10.3389/fphar.2021.755768)

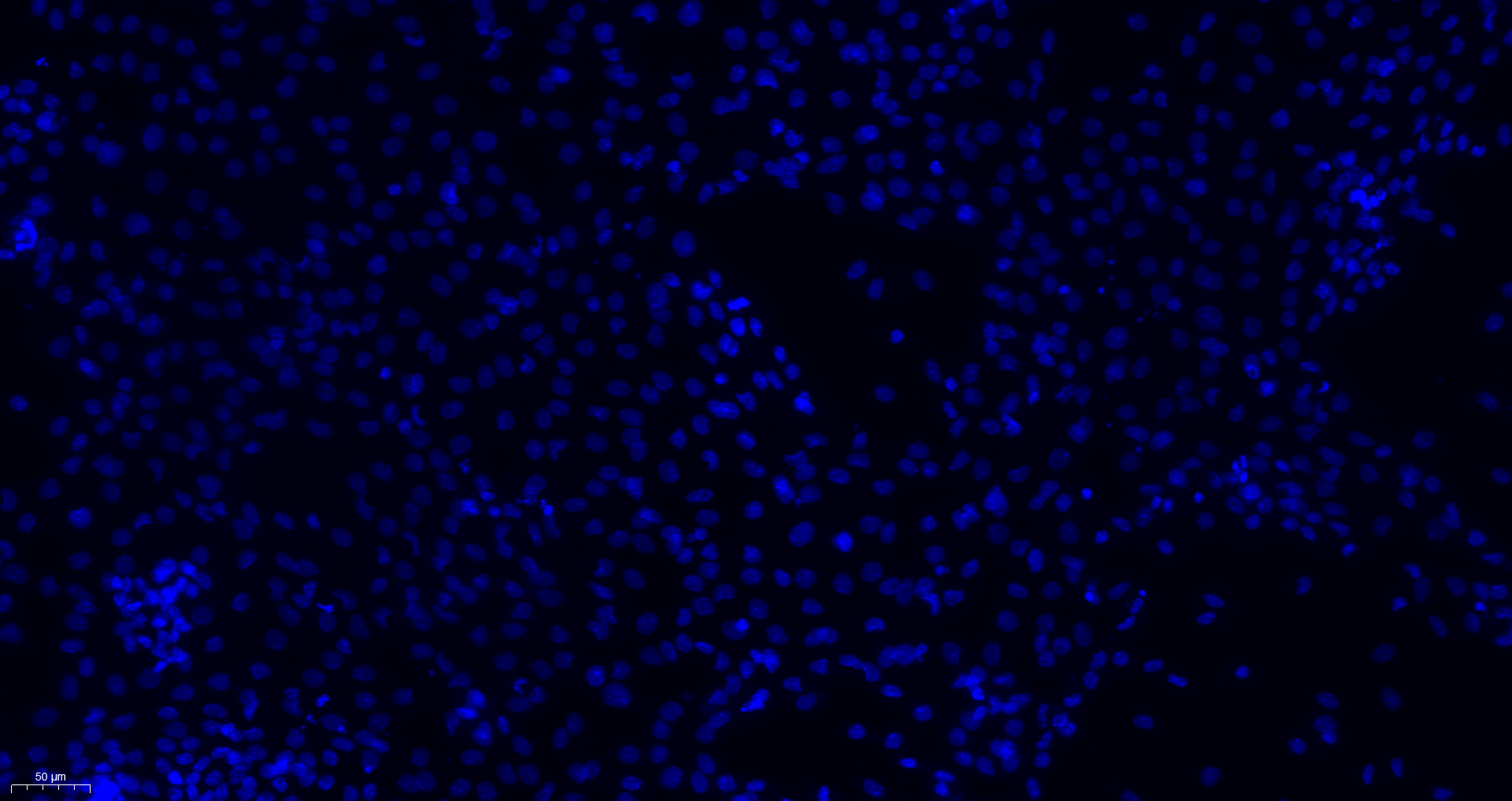

Supplement: Supplementary file 1 [file DataSheet1.ZIP › IF_the raw data of figure 7A,B/wnt3a┴/1W IF wnt3a┴║∞_20.0x 1.jpg]

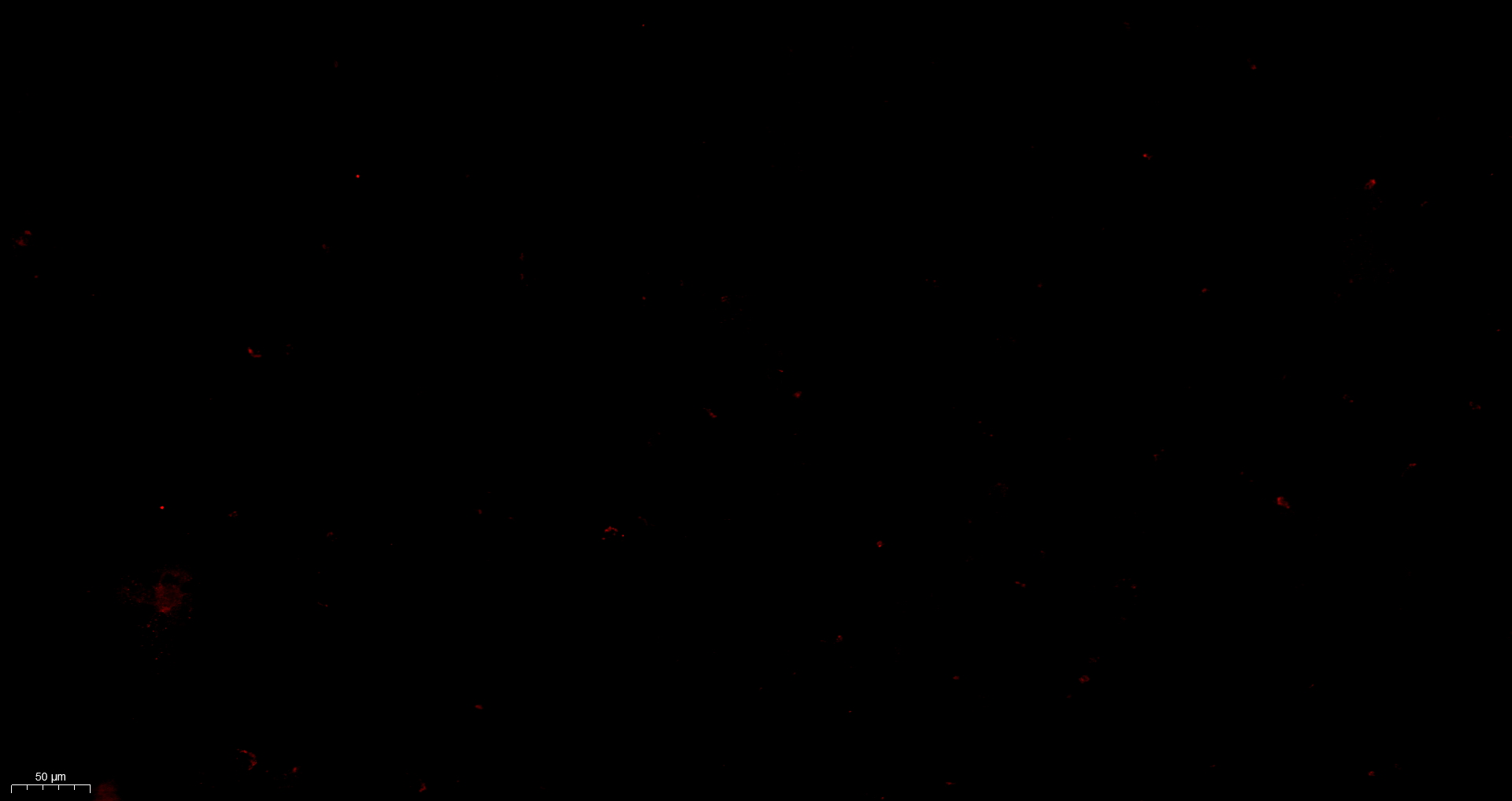

Supplement: Supplementary file 1 [file DataSheet1.ZIP › IF_the raw data of figure 7A,B/wnt3a┴/1W IF wnt3a┴║∞_20.0x 2.jpg]

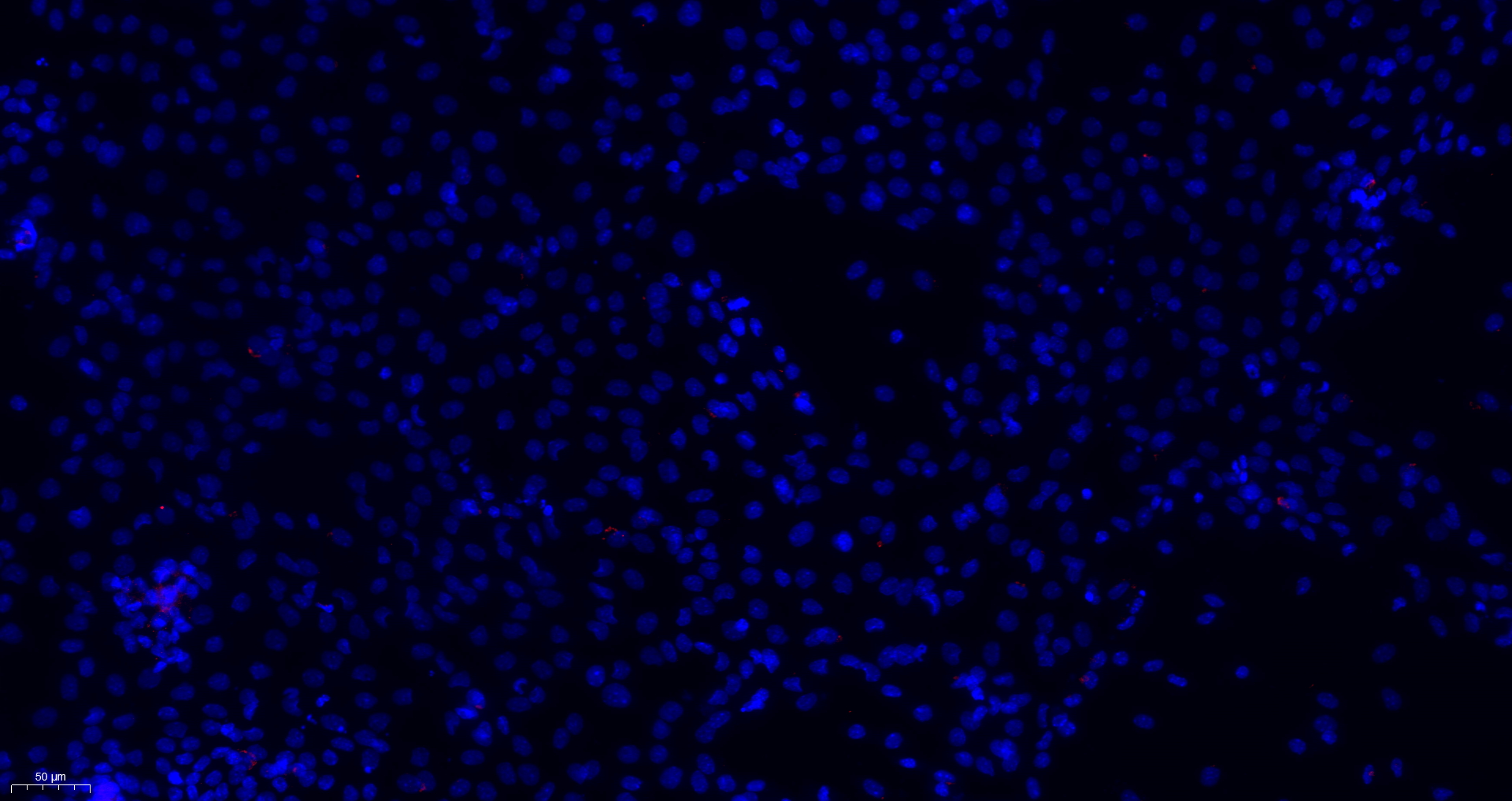

Supplement: Supplementary file 1 [file DataSheet1.ZIP › IF_the raw data of figure 7A,B/wnt3a┴/1W IF wnt3a┴║∞_20.0x 3.jpg]

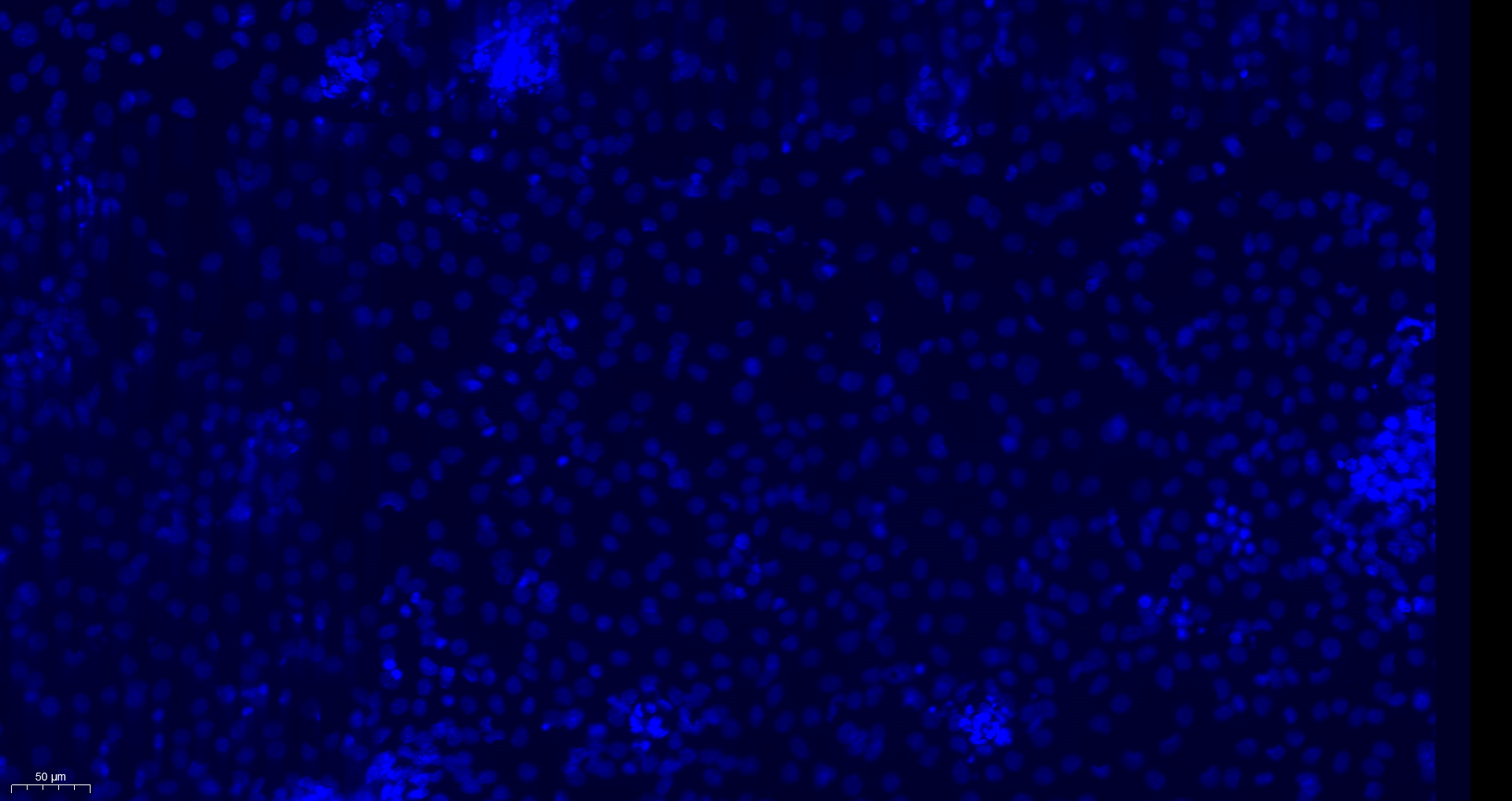

Supplement: Supplementary file 1 [file DataSheet1.ZIP › IF_the raw data of figure 7A,B/wnt3a┴/2W IF wnt3a┴║∞_20.0x 1.jpg]

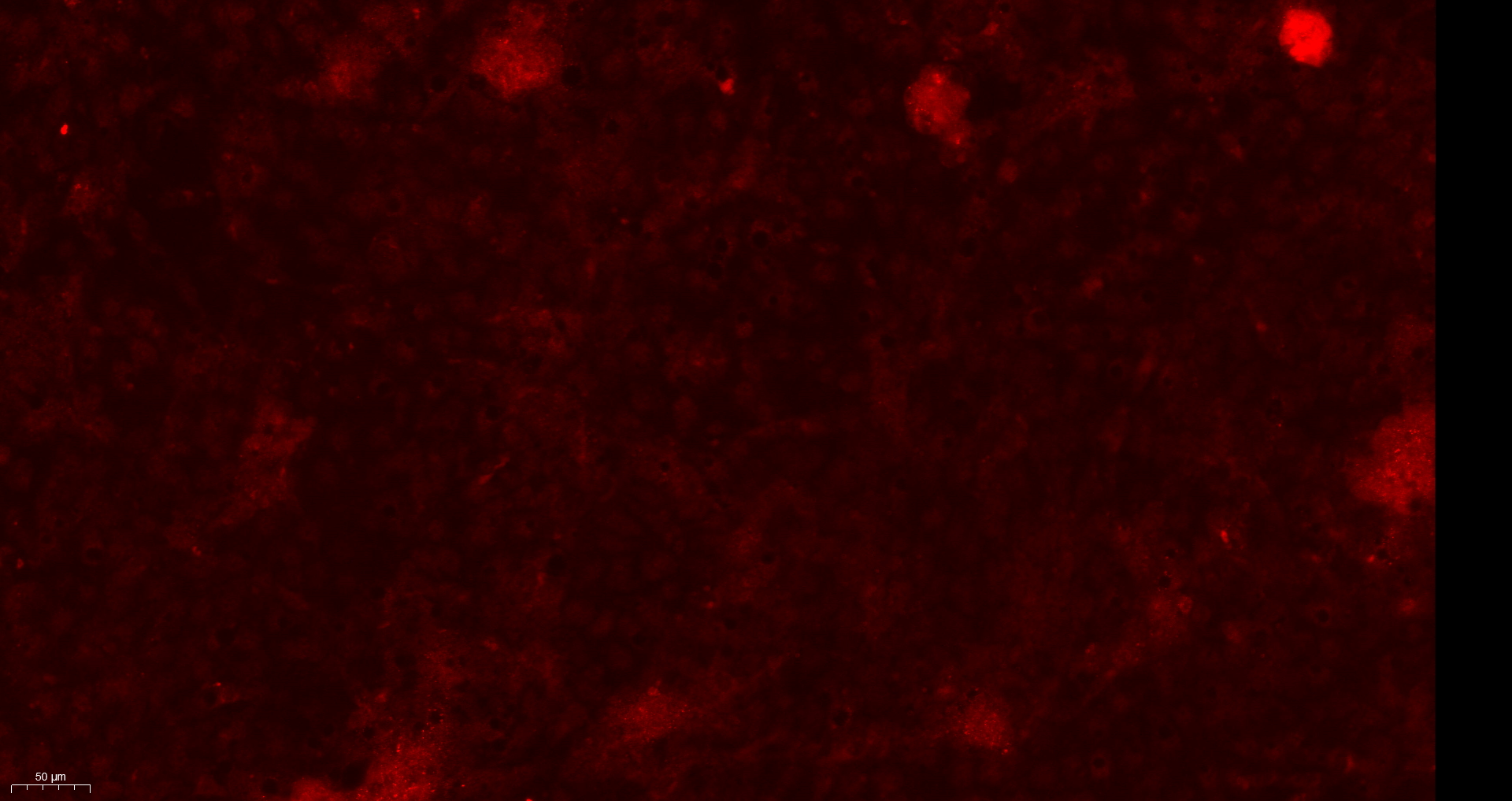

Supplement: Supplementary file 1 [file DataSheet1.ZIP › IF_the raw data of figure 7A,B/wnt3a┴/2W IF wnt3a┴║∞_20.0x 2.jpg]

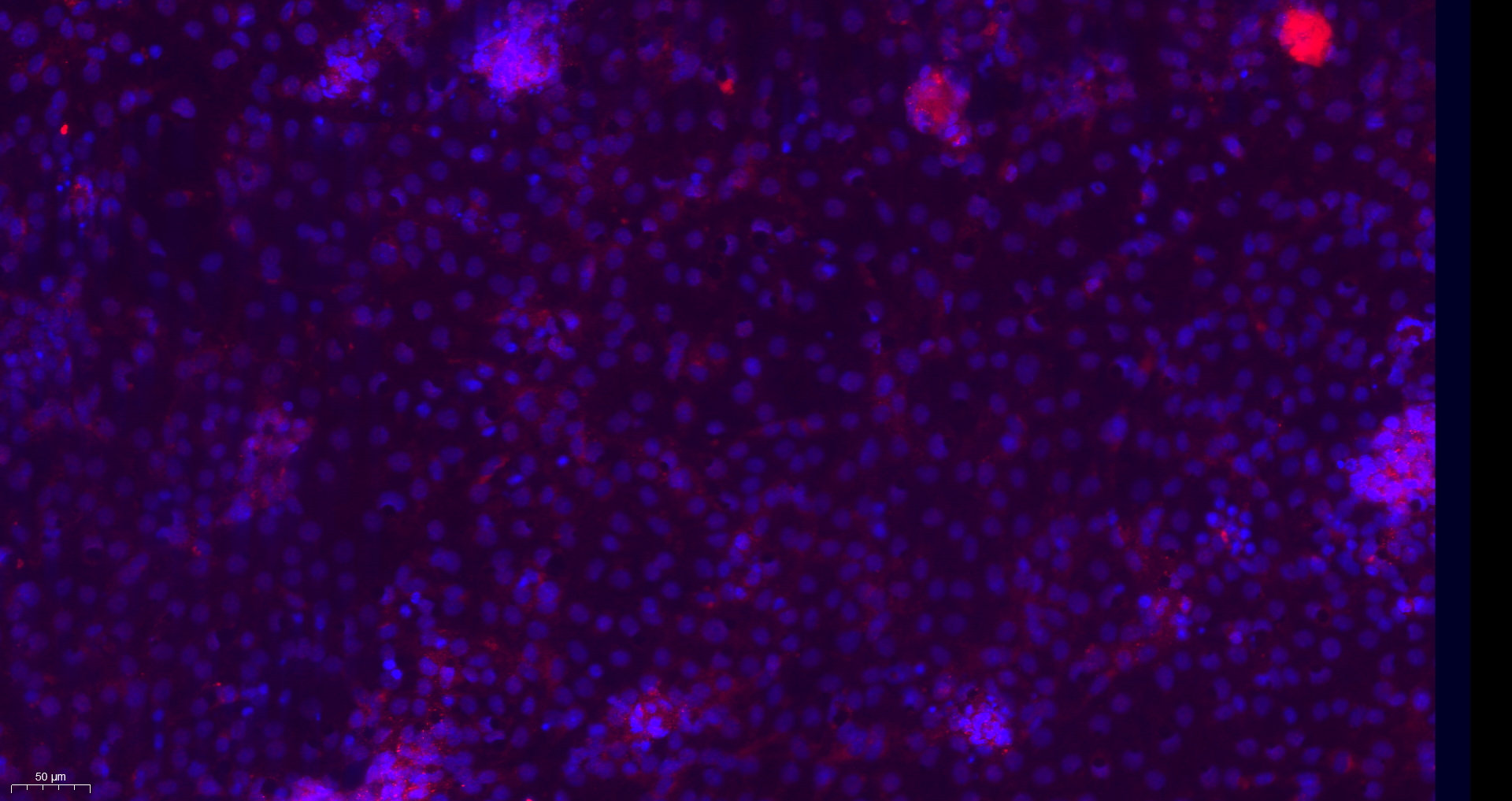

Supplement: Supplementary file 1 [file DataSheet1.ZIP › IF_the raw data of figure 7A,B/wnt3a┴/2W IF wnt3a┴║∞_20.0x 3.jpg]

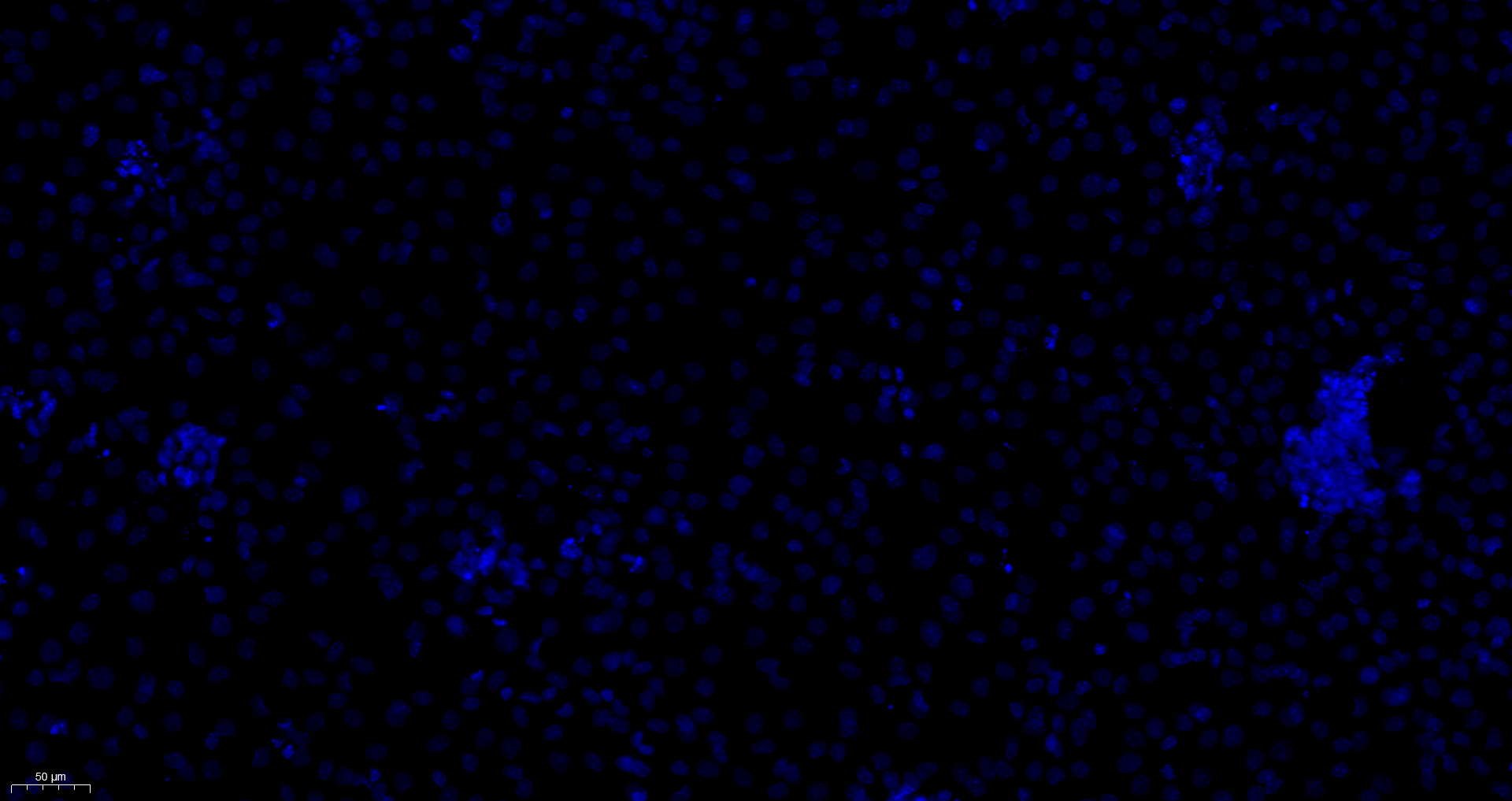

Supplement: Supplementary file 1 [file DataSheet1.ZIP › IF_the raw data of figure 7A,B/wnt3a┴/3W IF wnt3a┴║∞_20.0x 1.jpg]

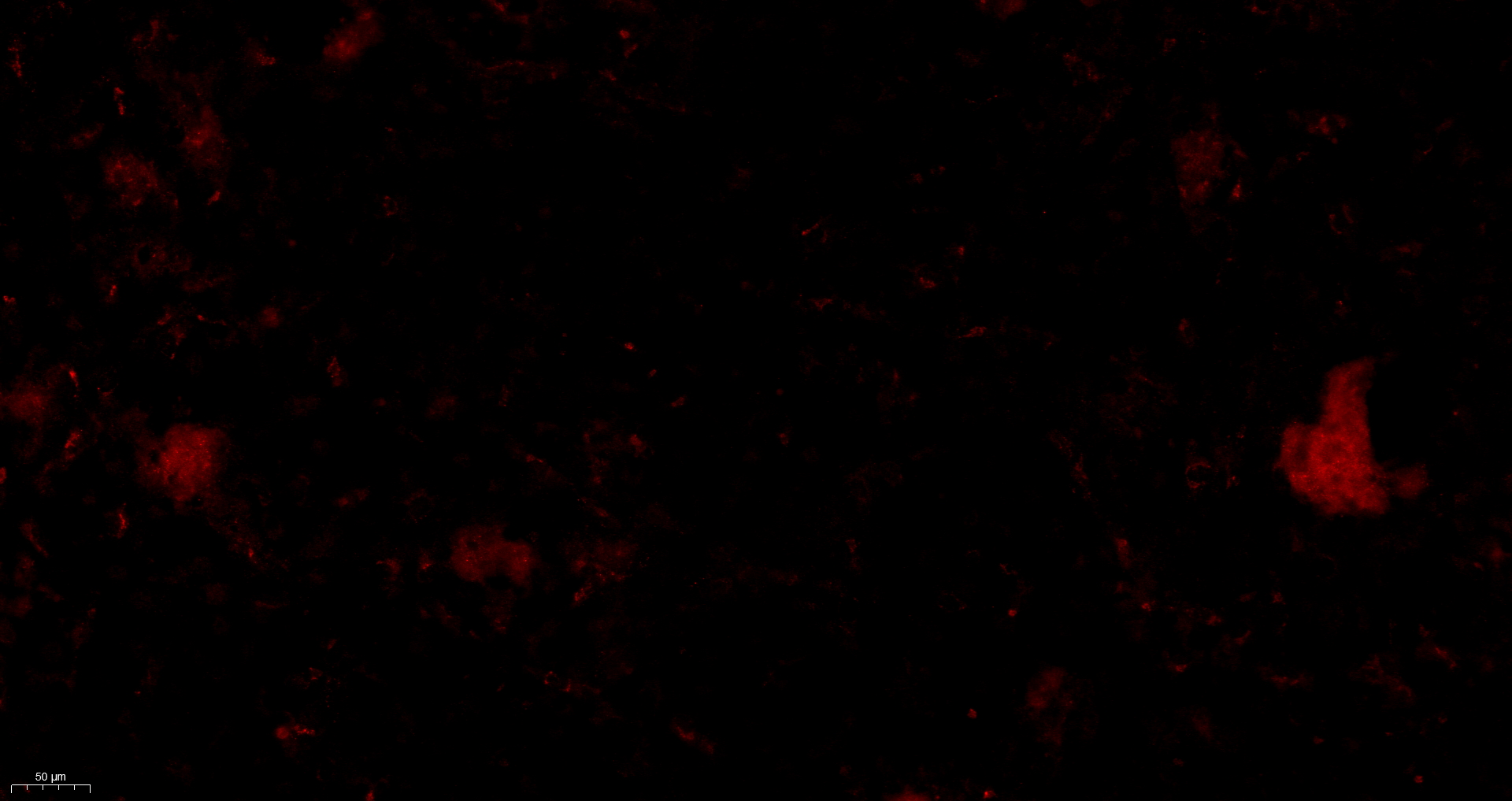

Supplement: Supplementary file 1 [file DataSheet1.ZIP › IF_the raw data of figure 7A,B/wnt3a┴/3W IF wnt3a┴║∞_20.0x 2.jpg]

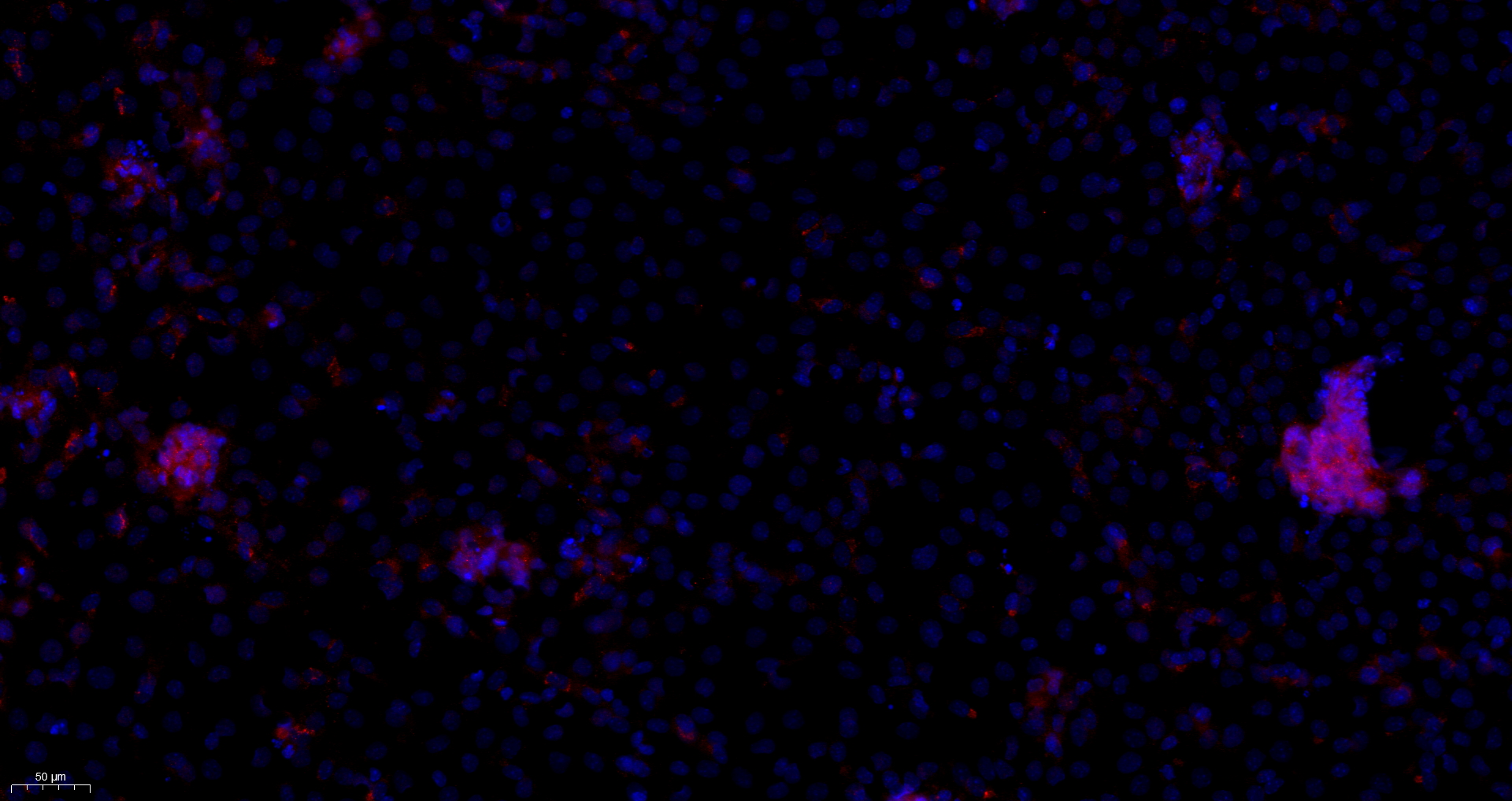

Supplement: Supplementary file 1 [file DataSheet1.ZIP › IF_the raw data of figure 7A,B/wnt3a┴/3W IF wnt3a┴║∞_20.0x 3.jpg]

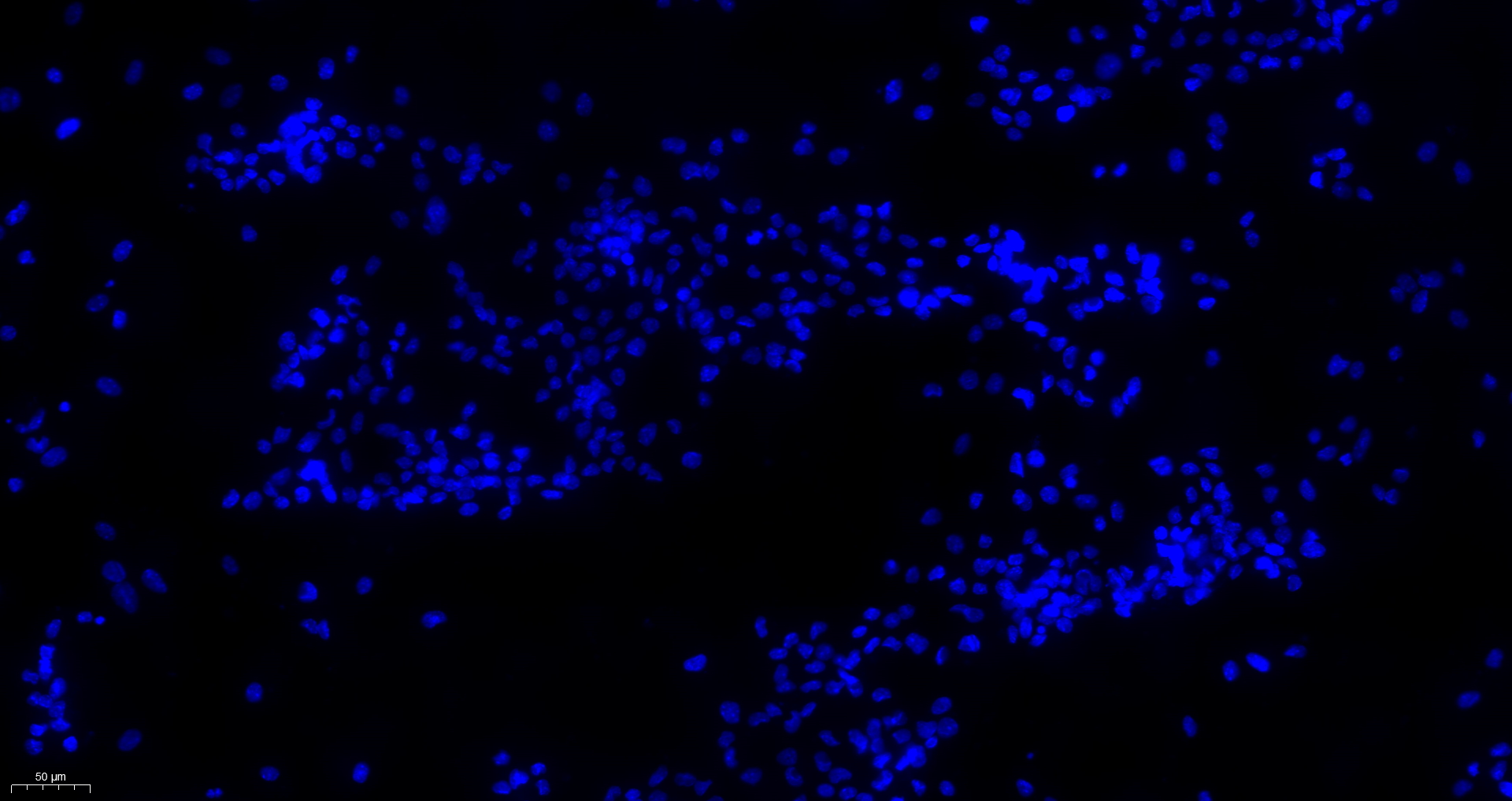

Supplement: Supplementary file 1 [file DataSheet1.ZIP › IF_the raw data of figure 7A,B/wnt3a┴/4W IF wnt3a┴║∞_20.0x 1.jpg]

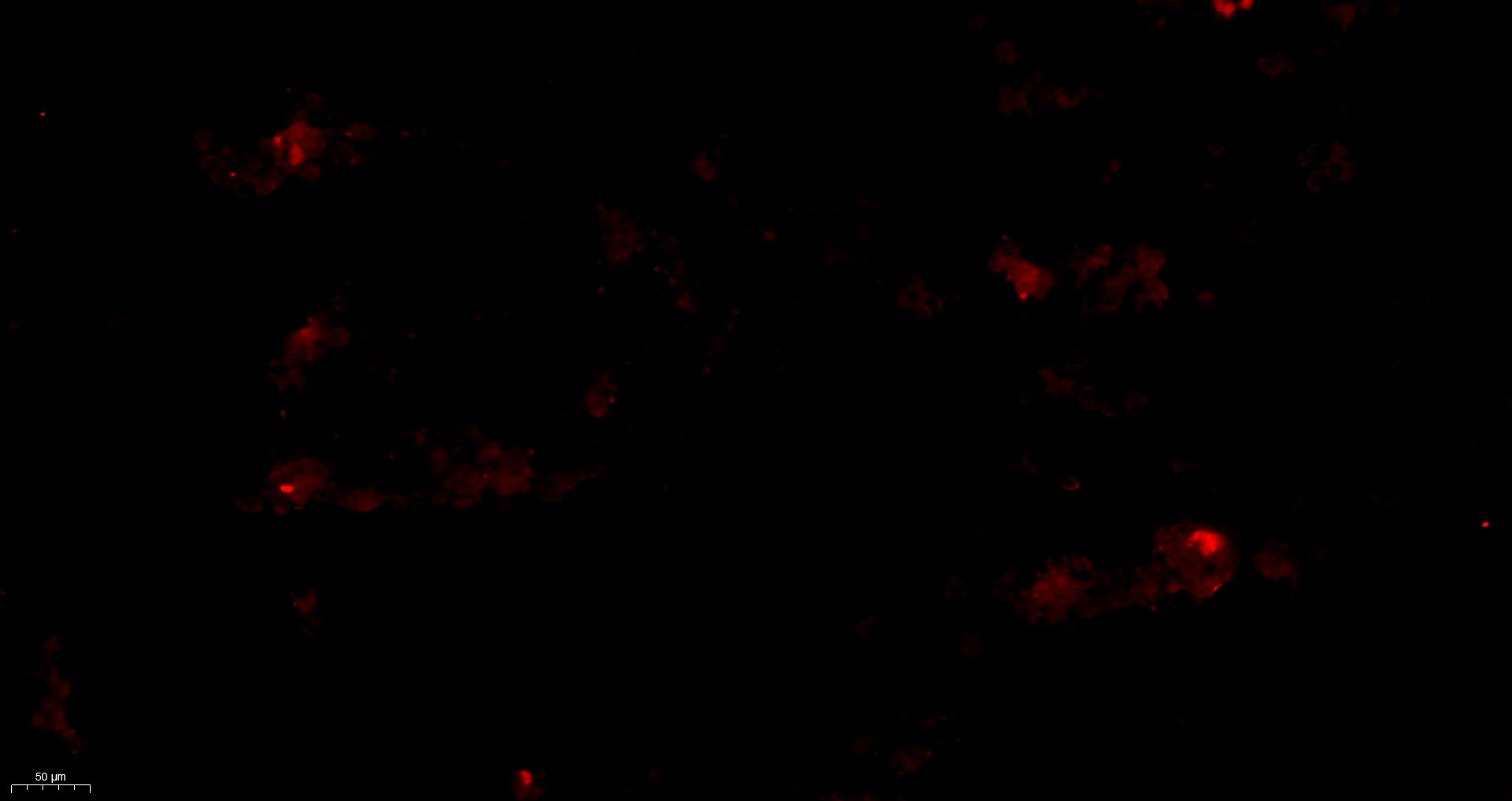

Supplement: Supplementary file 1 [file DataSheet1.ZIP › IF_the raw data of figure 7A,B/wnt3a┴/4W IF wnt3a┴║∞_20.0x 2.jpg]

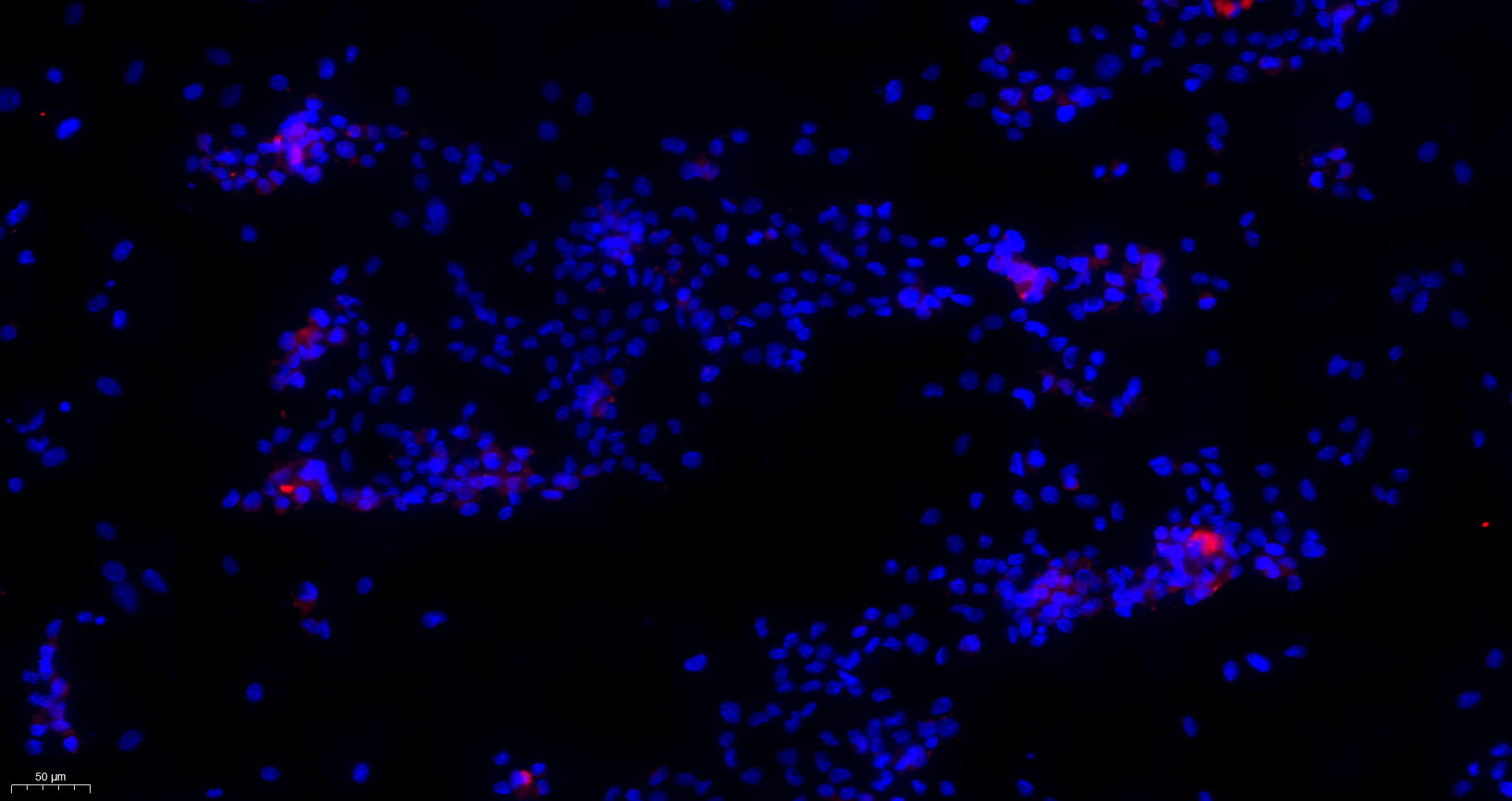

Supplement: Supplementary file 1 [file DataSheet1.ZIP › IF_the raw data of figure 7A,B/wnt3a┴/4W IF wnt3a┴║∞_20.0x 3.jpg]

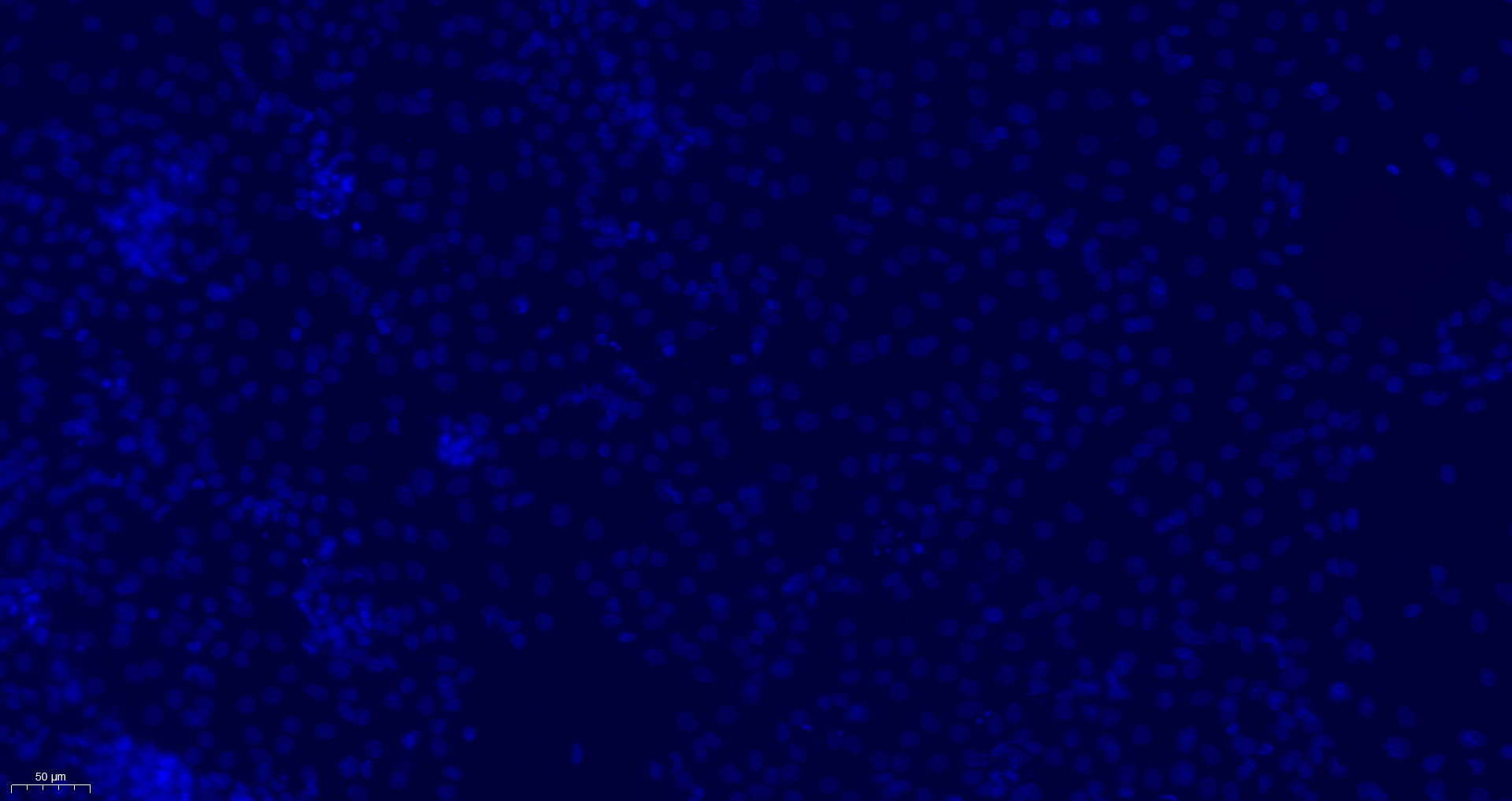

Supplement: Supplementary file 1 [file DataSheet1.ZIP › IF_the raw data of figure 7A,B/a┬-catenin/1B IF a┬-catenin┬╠_20.0x 1.jpg]

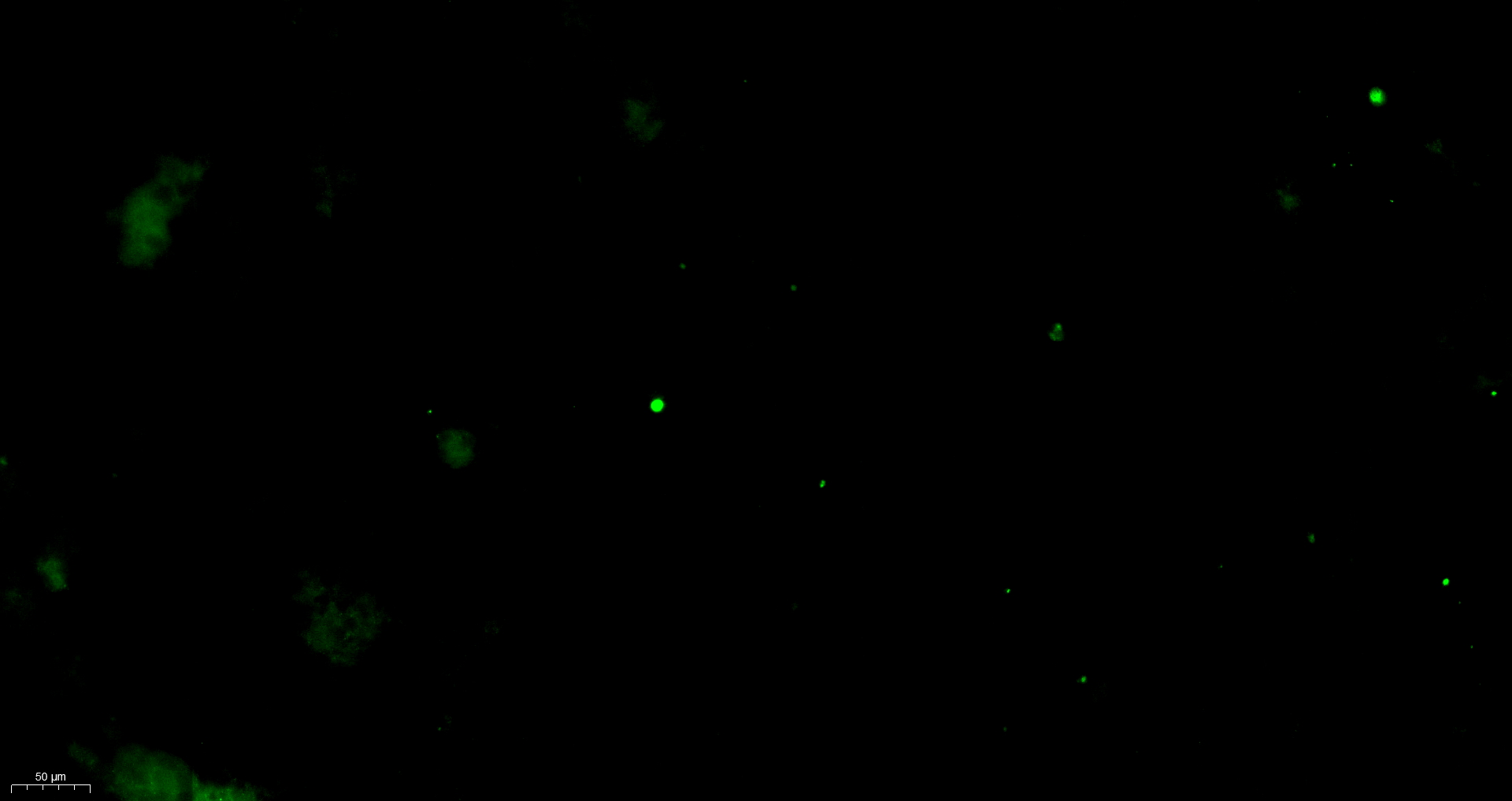

Supplement: Supplementary file 1 [file DataSheet1.ZIP › IF_the raw data of figure 7A,B/a┬-catenin/1B IF a┬-catenin┬╠_20.0x 2.jpg]

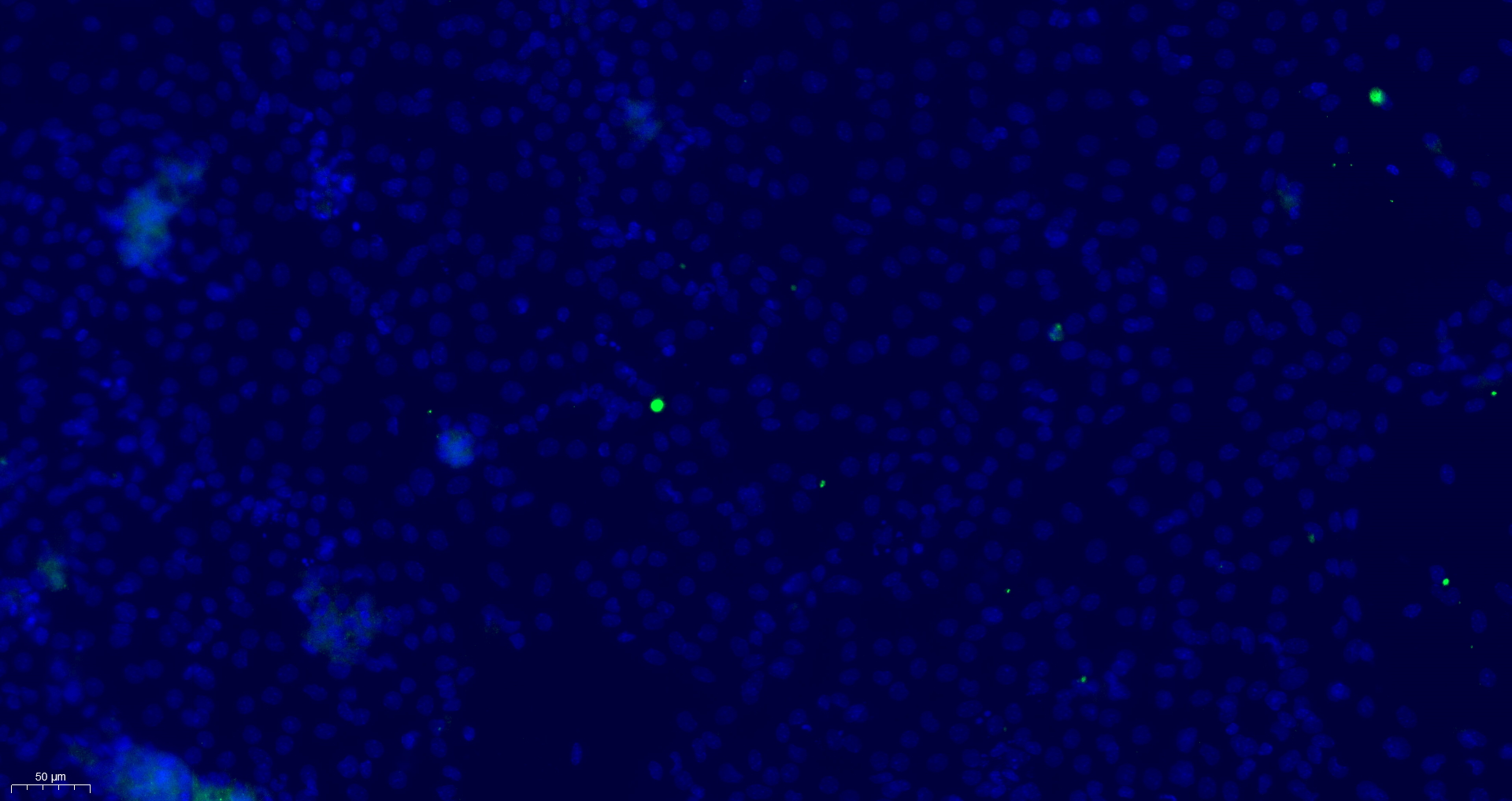

Supplement: Supplementary file 1 [file DataSheet1.ZIP › IF_the raw data of figure 7A,B/a┬-catenin/1B IF a┬-catenin┬╠_20.0x 3.jpg]

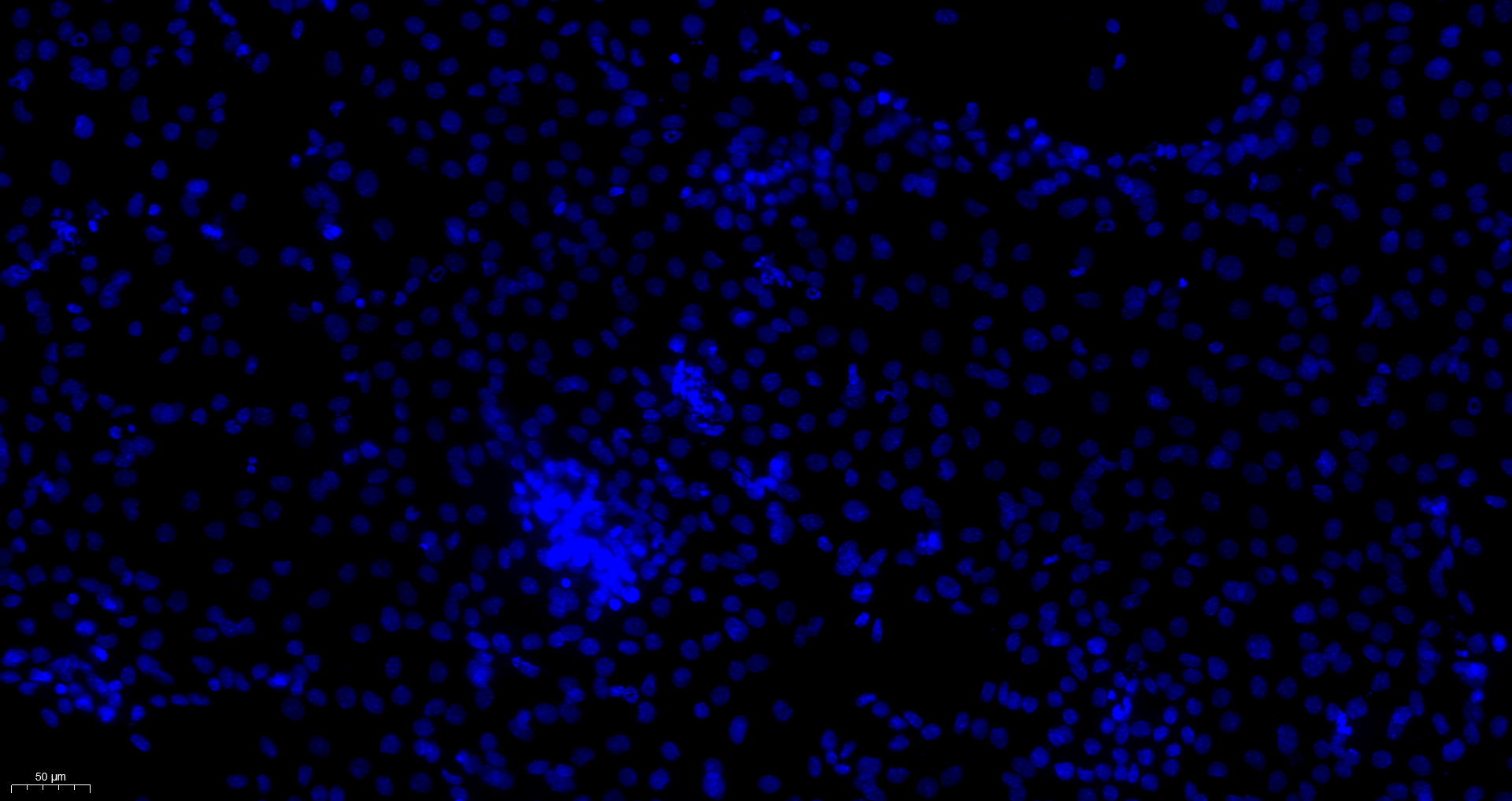

Supplement: Supplementary file 1 [file DataSheet1.ZIP › IF_the raw data of figure 7A,B/a┬-catenin/2B IF a┬-catenin┬╠_20.0x 1.jpg]

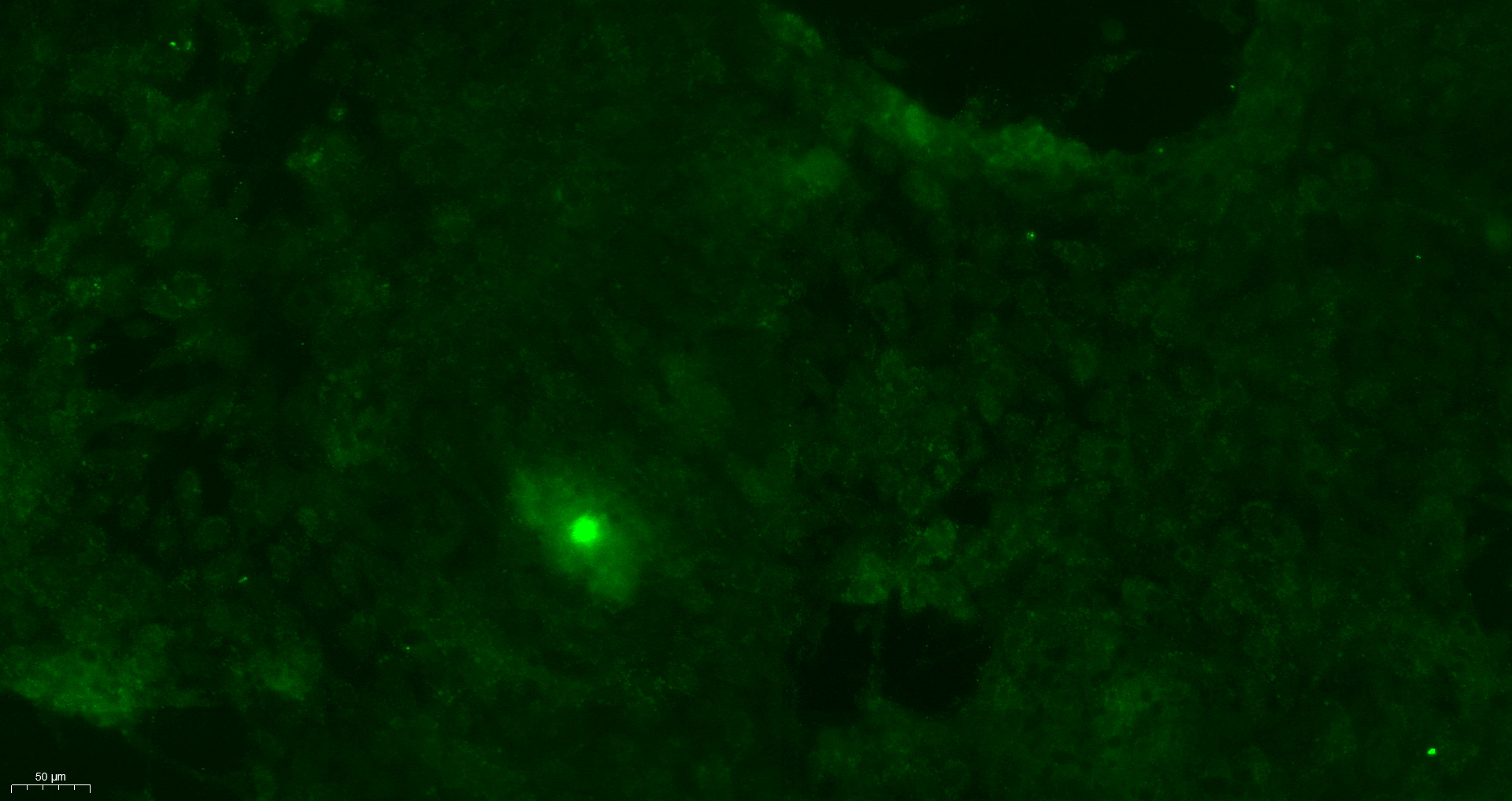

Supplement: Supplementary file 1 [file DataSheet1.ZIP › IF_the raw data of figure 7A,B/a┬-catenin/2B IF a┬-catenin┬╠_20.0x 2.jpg]

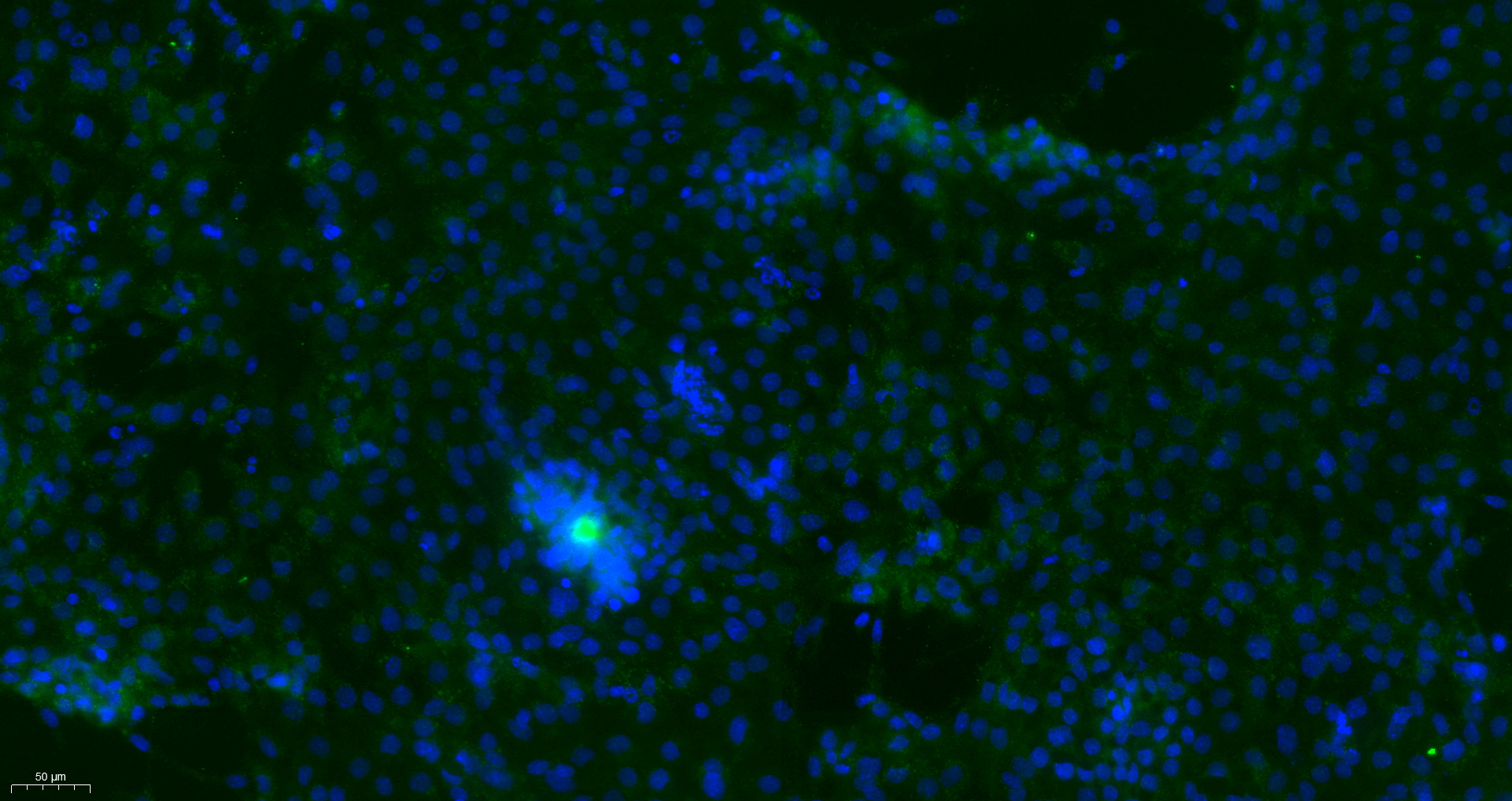

Supplement: Supplementary file 1 [file DataSheet1.ZIP › IF_the raw data of figure 7A,B/a┬-catenin/2B IF a┬-catenin┬╠_20.0x 3.jpg]

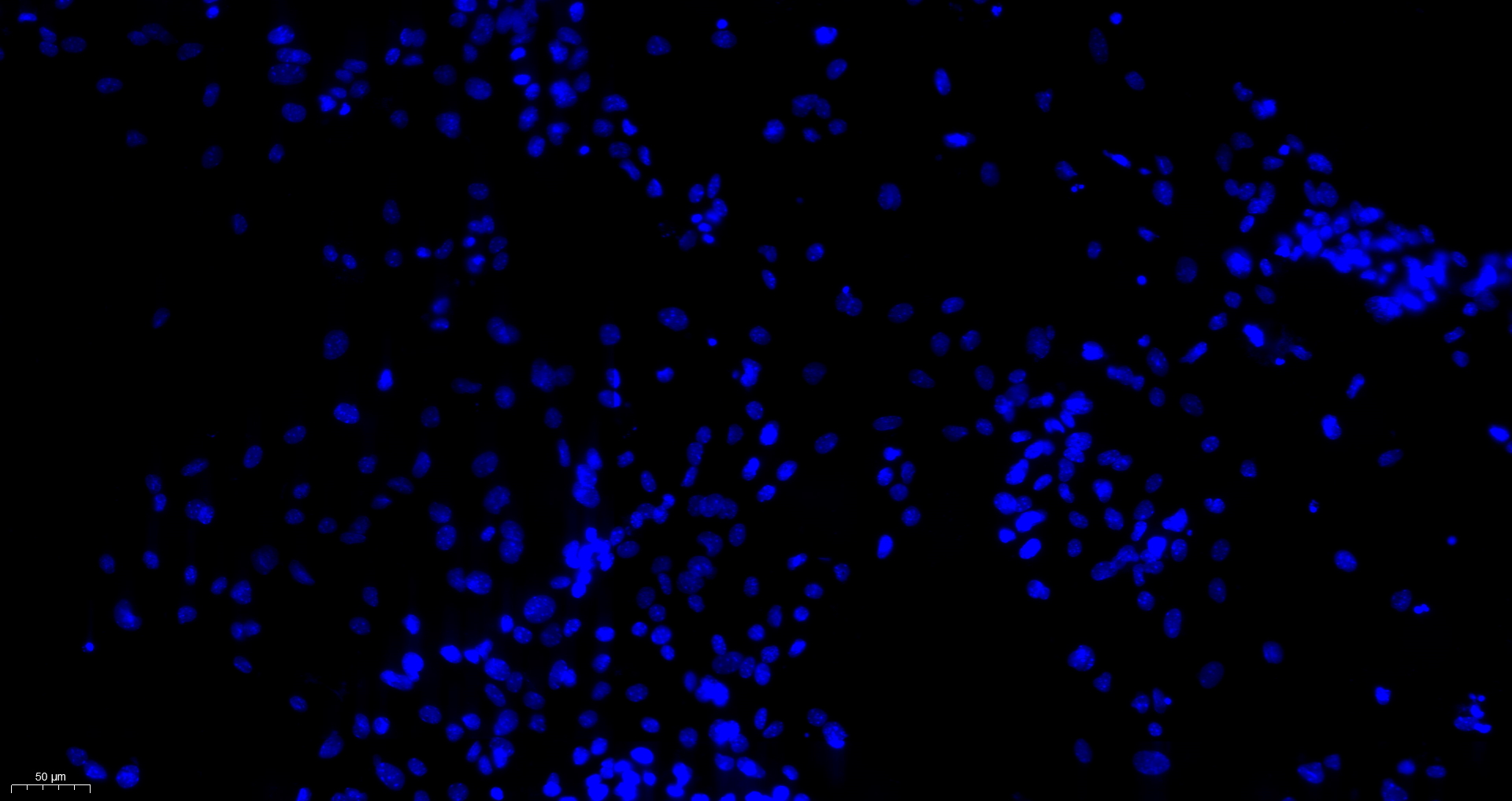

Supplement: Supplementary file 1 [file DataSheet1.ZIP › IF_the raw data of figure 7A,B/a┬-catenin/3B IF a┬-catenin_20.0x 1.jpg]

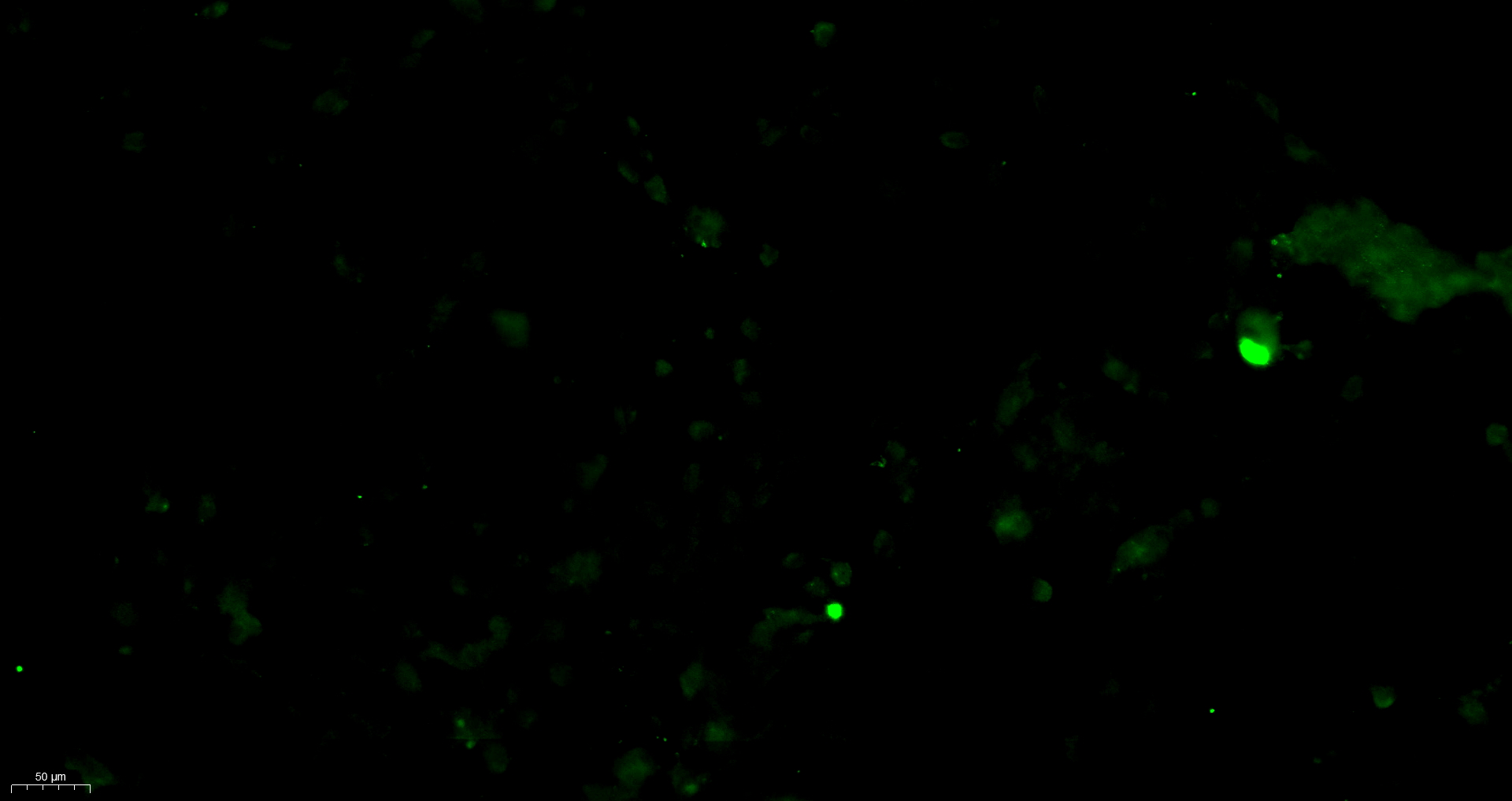

Supplement: Supplementary file 1 [file DataSheet1.ZIP › IF_the raw data of figure 7A,B/a┬-catenin/3B IF a┬-catenin┬╠_20.0x 2.jpg]

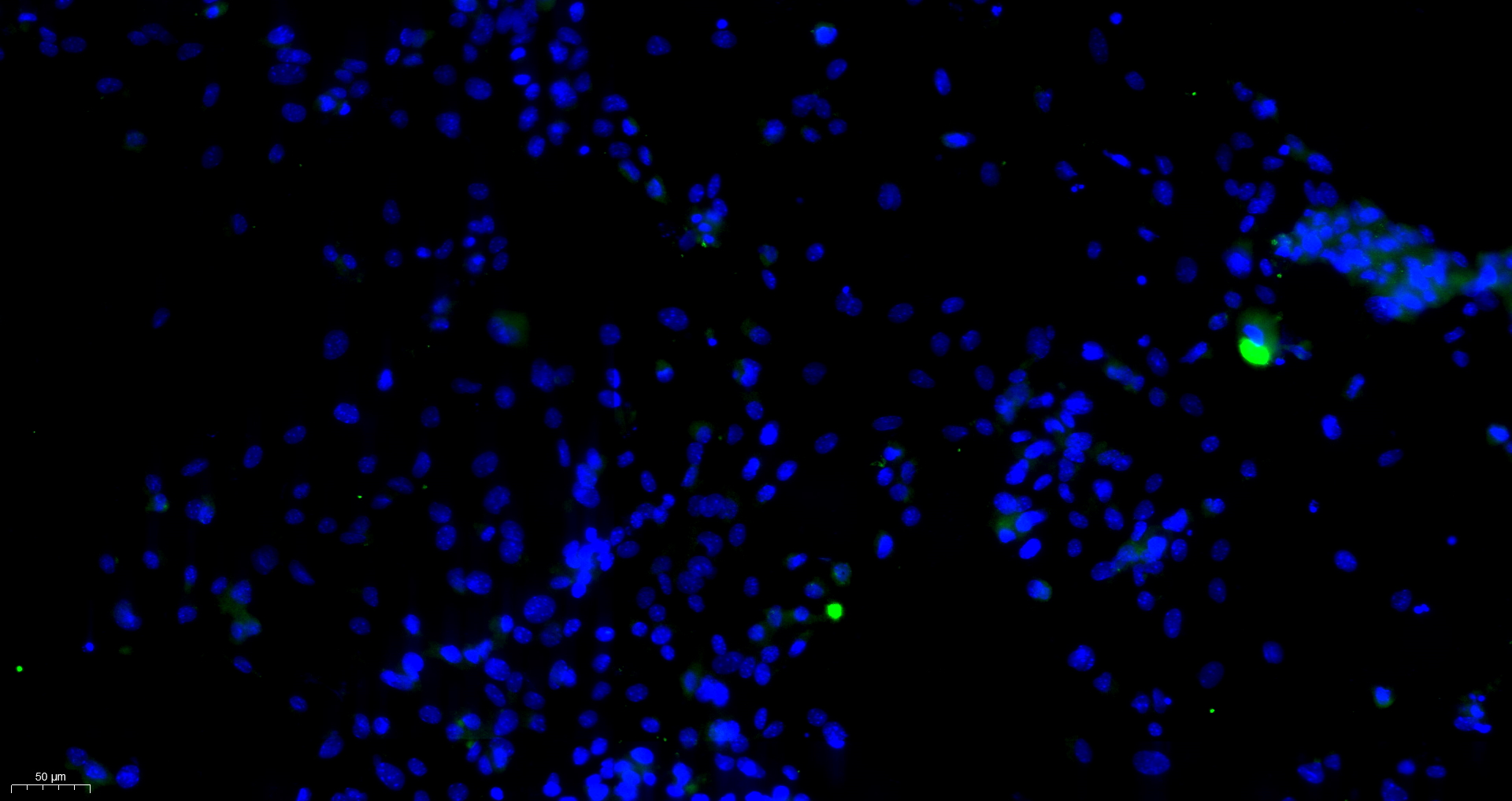

Supplement: Supplementary file 1 [file DataSheet1.ZIP › IF_the raw data of figure 7A,B/a┬-catenin/3B IF a┬-catenin┬╠_20.0x 3.jpg]

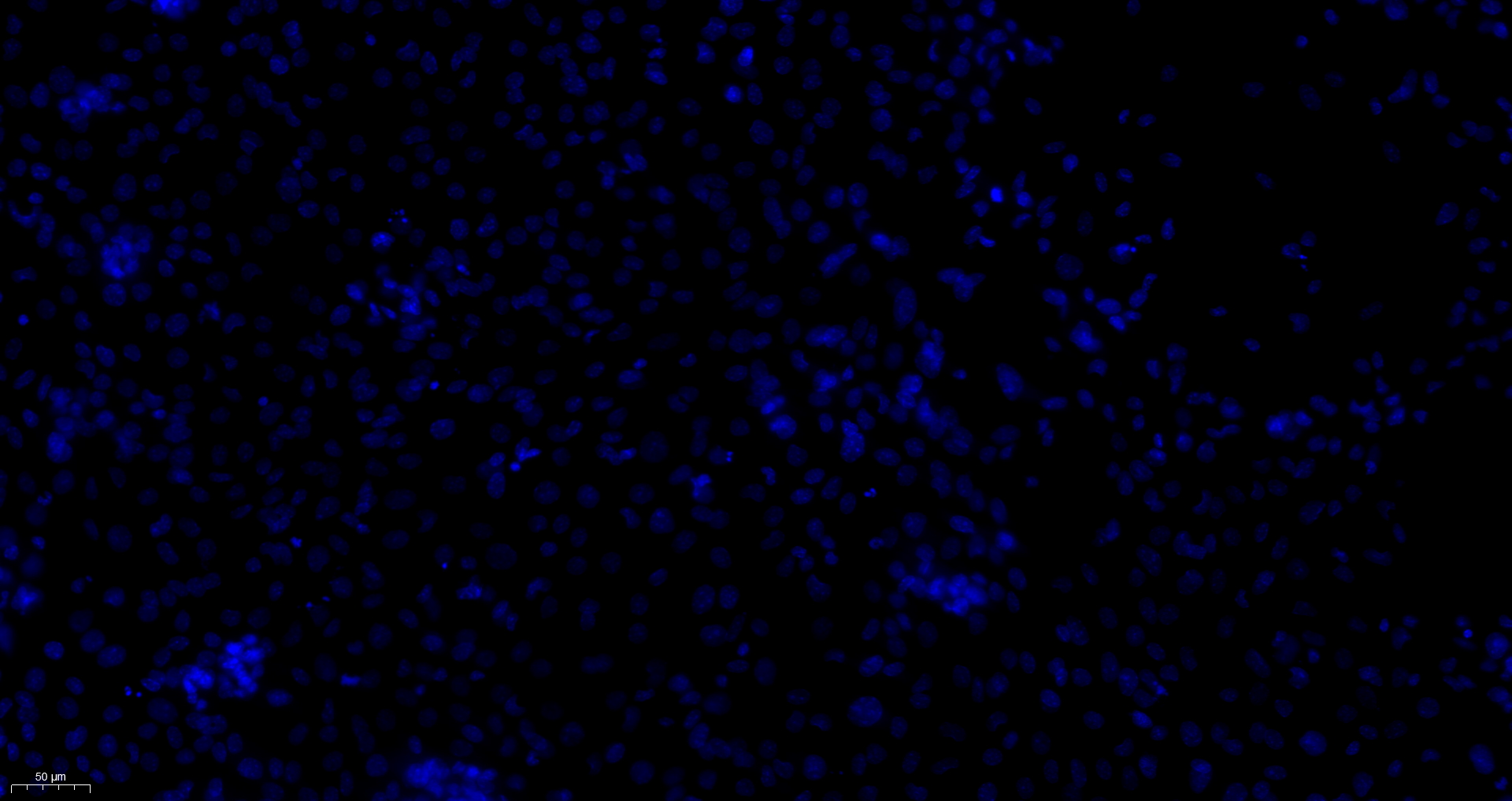

Supplement: Supplementary file 1 [file DataSheet1.ZIP › IF_the raw data of figure 7A,B/a┬-catenin/4B IF a┬-catenin┬╠_20.0x 1.jpg]

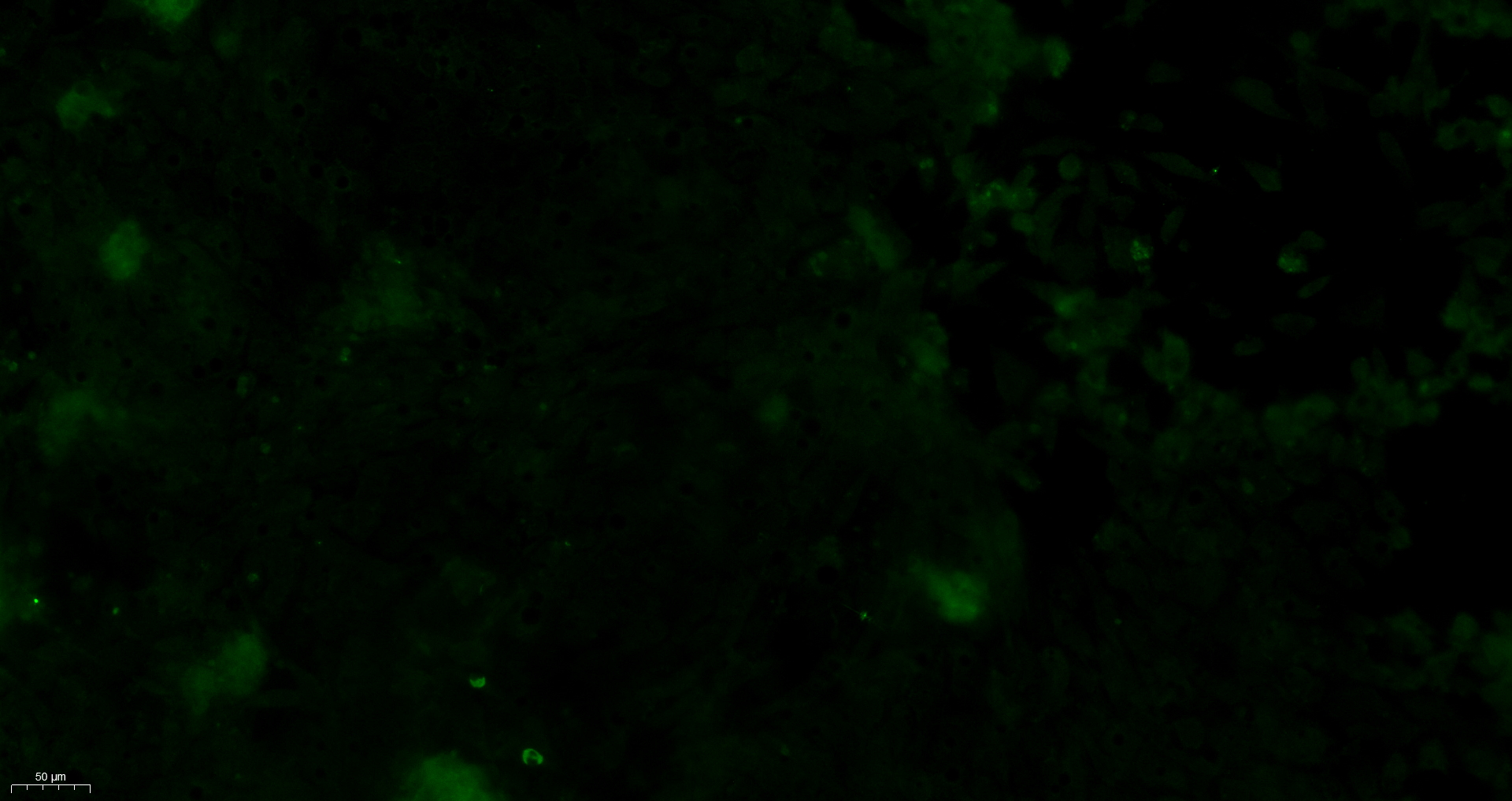

Supplement: Supplementary file 1 [file DataSheet1.ZIP › IF_the raw data of figure 7A,B/a┬-catenin/4B IF a┬-catenin┬╠_20.0x 2.jpg]

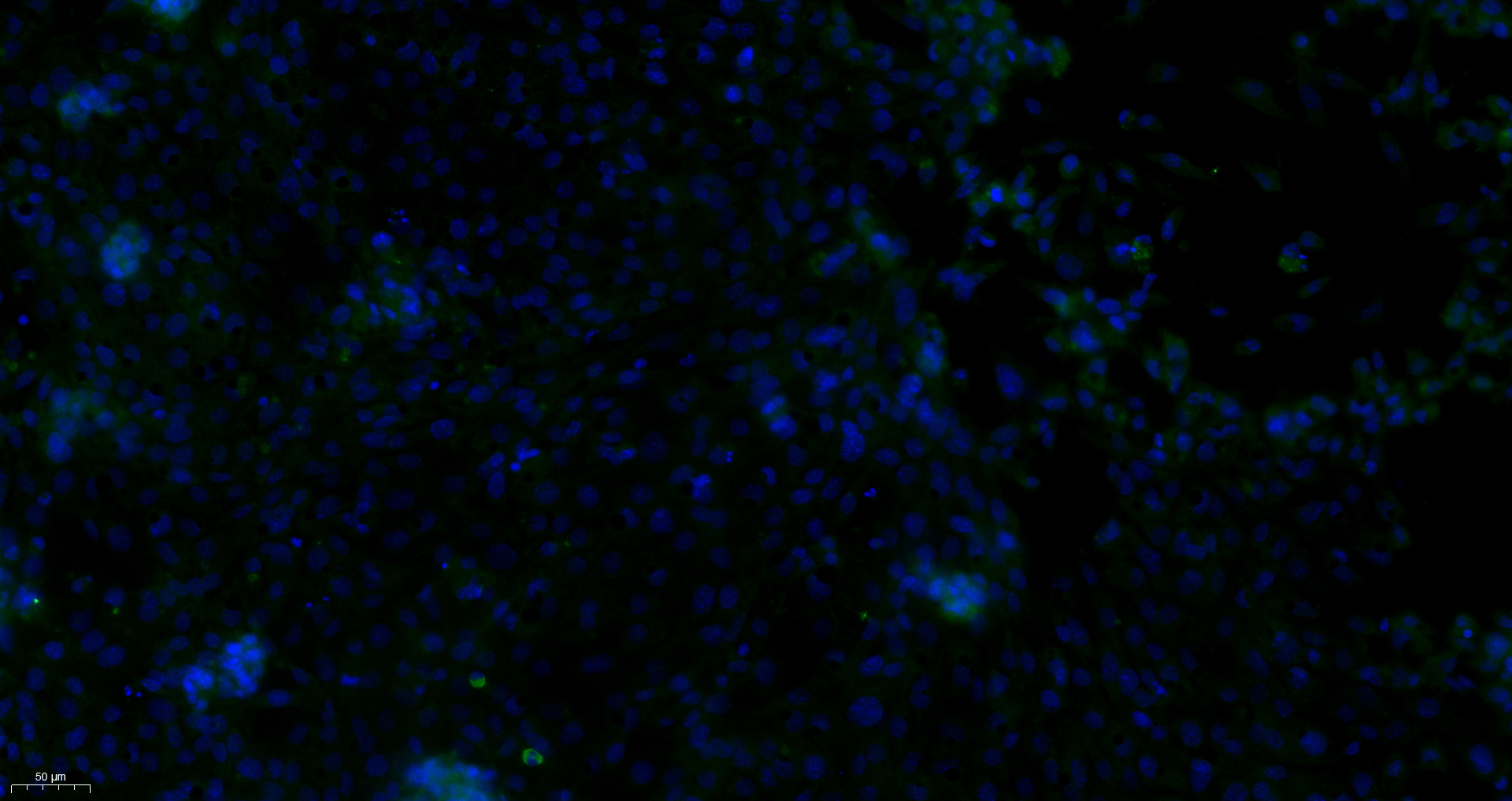

Supplement: Supplementary file 1 [file DataSheet1.ZIP › IF_the raw data of figure 7A,B/a┬-catenin/4B IF a┬-catenin┬╠_20.0x 3.jpg]

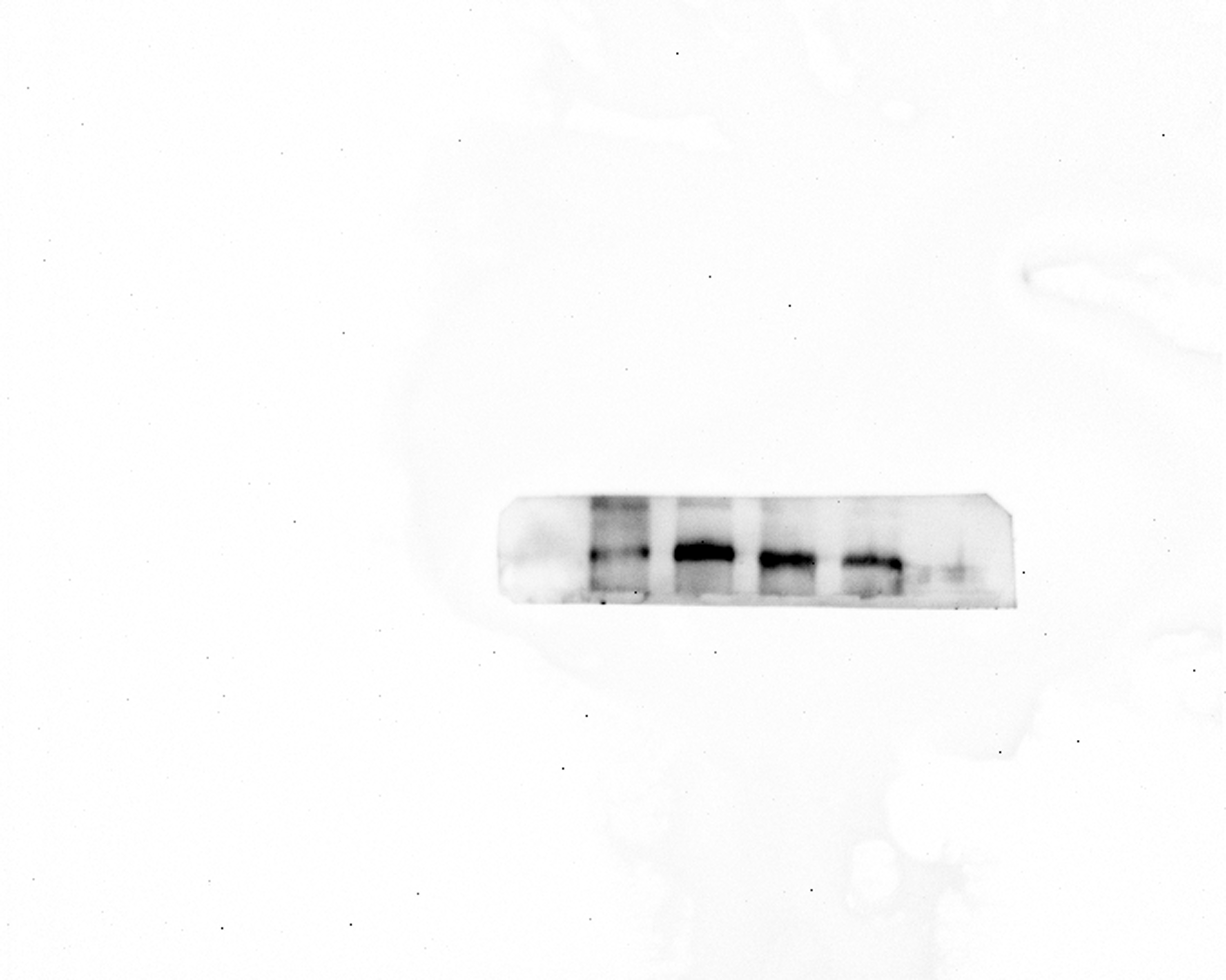

Supplement: Supplementary file 2 [file DataSheet2.ZIP › proteins_the raw data of figure 7C/Wnt3a┴.tif]

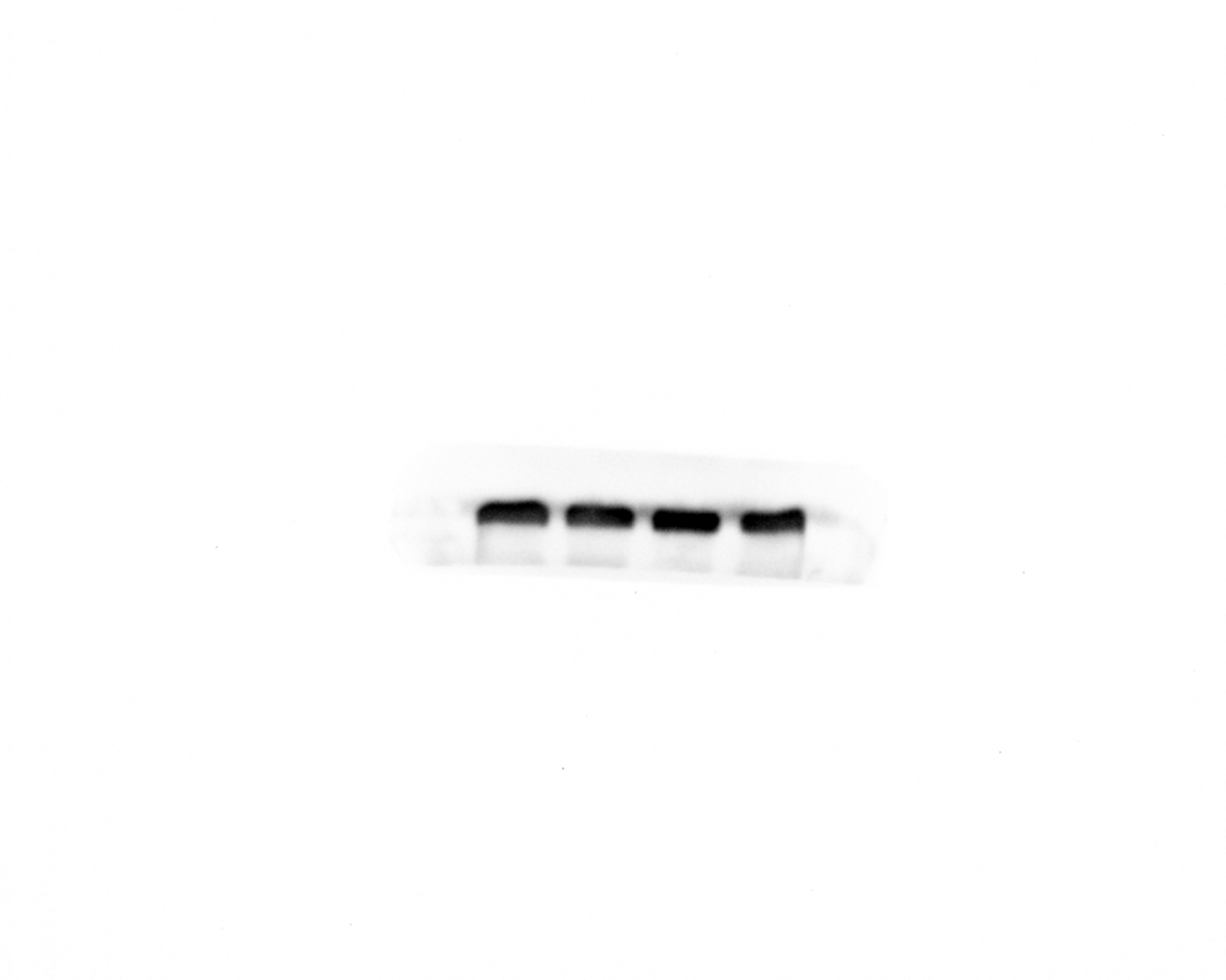

Supplement: Supplementary file 2 [file DataSheet2.ZIP › proteins_the raw data of figure 7C/a┬-actin.tif]

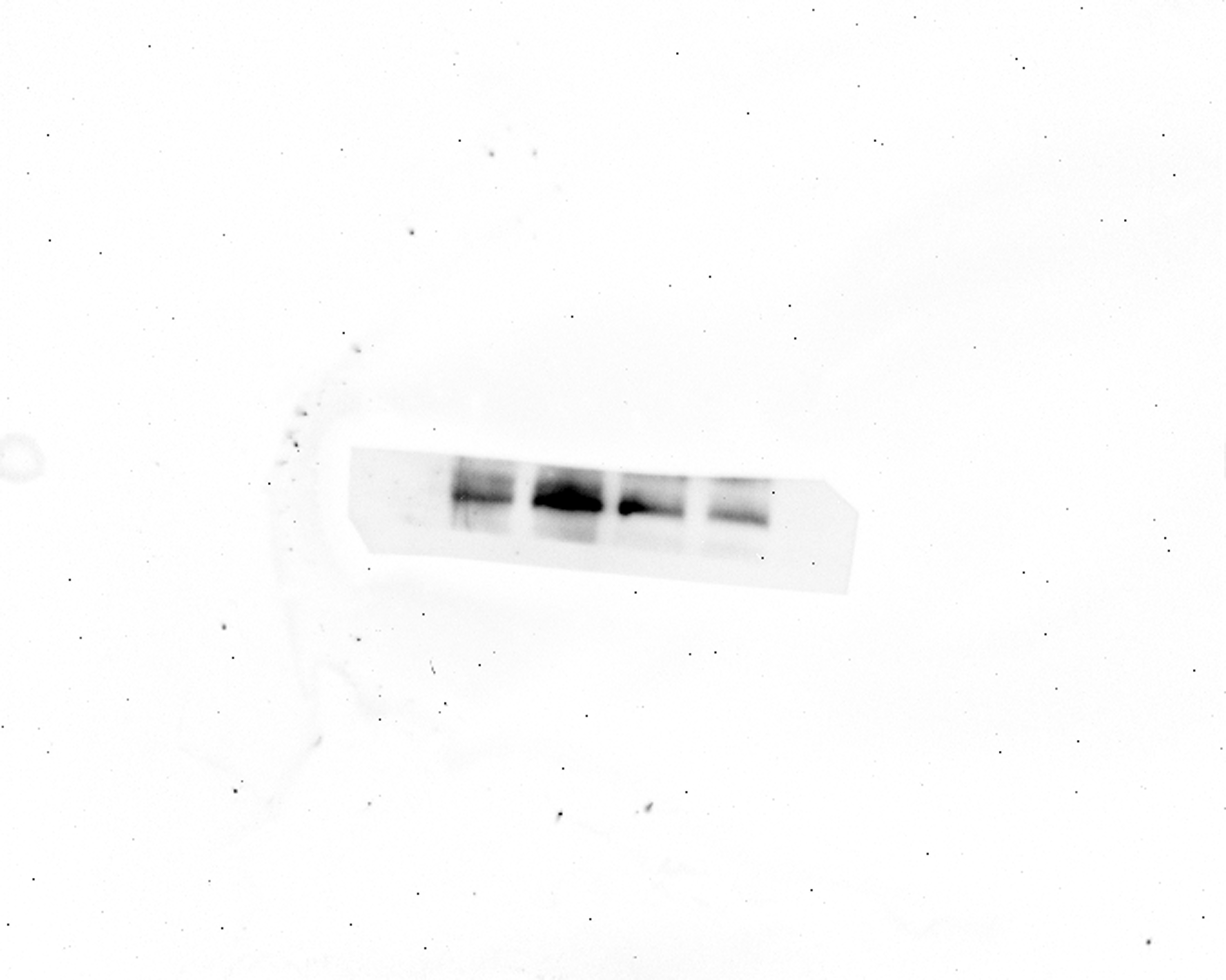

Supplement: Supplementary file 2 [file DataSheet2.ZIP › proteins_the raw data of figure 7C/a┬-catenin.tif]
